# Development, internal and external evaluation of an artificial intelligence algorithm for child growth monitoring in primary care

**DOI:** 10.1371/journal.pdig.0001526

**Published:** 2026-07-15

**Authors:** Pauline Scherdel, Raphaële Houlbracq, Emmanuel Lecoeur, Juliane Léger, Raja Brauner, Jérémie F. Cohen, EBGM V Study Group, Martin Chalumeau, Barbara Heude

**Affiliations:** 1 Université Paris Cité and Université Sorbonne Paris Nord, Inserm, INRAE, Centre for Research in Epidemiology and StatisticS (CRESS), Paris, France; 2 Université Paris Cité, Inserm, UMR1343, Pharmacologie et Évaluations des Thérapeutiques Chez l’enfant et la Femme Enceinte, Paris, France; 3 Unité de Recherche Clinique, Université Paris Cité Necker/Cochin, Paris, France; 4 Department of Pediatric Endocrinology and Diabetology, Robert-Debré Hospital, AP-HP, Université Paris Cité, Paris, France; 5 Reference Centre for Endocrine Growth and Development Diseases, Paris, France; 6 Unité d’Endocrinologie Pédiatrique, Hôpital Fondation Adolphe de Rothschild, Paris, France; 7 Department of General Pediatrics and Pediatric Infectious Diseases, Necker-Enfants Malades Hospital, AP-HP, Université Paris Cité, Paris, France; Liverpool John Moores University - City Campus: Liverpool John Moores University, UNITED KINGDOM OF GREAT BRITAIN AND NORTHERN IRELAND

## Abstract

Our goal was to improve growth monitoring in children by developing and evaluating an artificial intelligence (AI) algorithm that can detect abnormal growth. We used pre-diagnosis height measurements for children with a diagnosis of growth hormone deficiency (GHD, n = 86) or Turner syndrome (TS, n = 87) in France (1990–2014) and all height measurements of apparently healthy children (referents, n = 923). We modeled the individual height growth curves by applying non-linear mixed models for each new measurement of each child. The resulting growth parameters were used in multinomial logistic regression across five pre-defined age ranges from 1 to 12 years to predict abnormal growth trajectories. For the five age-specific predictive models, we studied the discrimination and calibration curves, and retained the risk thresholds that offered a pre-defined specificity of 98%. Using all the available height measurements for cases and referents, we evaluated the cumulative diagnostic performance of the algorithm for detecting GHD or TS and the theoretical reduction in time to diagnosis. We evaluated these models internally using 5-fold cross-validation and externally from a regional sample of children with GHD (n = 77) or TS (n = 40) and a national sample of apparently healthy children (n = 5,755). The five age-specific predictive models had good discrimination (high AUROC range 0.87-0.99) and good calibration. Internal evaluations showed stable results. External evaluation revealed a cumulative sensitivity and specificity of 84.6% (95% CI 76.8-90.6) and 94.3% (93.6-94.9). The median theoretical reduction in time to diagnosis was 2.0 years (interquartile range 0.6-3.8): 1.6 years (0.5-2.8) for GHD and 3.0 years (1.0-5.4) for TS. To conclude, we developed and internally and externally evaluated an AI algorithm with high diagnostic performance for the early detection of GHD and TS. A refinement of the algorithm to include other target conditions and further external evaluation in other countries is needed before implementation in daily practice.

## Author summary

Our goal was to improve growth monitoring in children by developing and evaluating an artificial intelligence (AI) algorithm that can detect abnormal growth. We used pre-diagnosis height measurements for children with a diagnosis of growth hormone deficiency (GHD, n = 86) or Turner syndrome (TS, n = 87) and all height measurements of apparently healthy children (referents, n = 923). We modeled the individual height growth curves by applying non-linear mixed models for each new measurement of each child. The resulting growth parameters were used in multinomial logistic regression across five pre-defined age ranges from 1 to 12 years to predict abnormal growth trajectories. For these age-specific predictive models, we achieved good discrimination and calibration, and retained the risk thresholds that offered a pre-defined specificity of 98%. We evaluated these age-specific models internally using 5-fold cross-validation and externally from a regional sample of children with GHD (n = 77) or TS (n = 40) and a national sample of apparently healthy children (n = 5,755). External evaluation revealed cumulative sensitivity and specificity of 84.6% (95% CI 76.8-90.6) and 94.3% (93.6-94.9). The median theoretical reduction in time to diagnosis was 2.0 years (interquartile range 0.6-3.8): 1.6 years (0.5-2.8) for GHD and 3.0 years (1.0-5.4) for TS. A refinement to include other target conditions is needed before implementation in daily practice.

## Introduction

Growth monitoring of children is a universal activity involving both clinical expertise and algorithms that define abnormal growth, aiming at detecting serious health conditions early and improving their prognosis [1]. For target conditions such as growth hormone deficiency (GHD), Turner syndrome (TS), celiac disease, Crohn’s disease, craniopharyngioma, hypothalamic-optochiasmatic tumors, and chronic kidney disease, substantial empirical evidence shows that current growth monitoring practices worldwide are suboptimal, notably in countries with advanced health systems [2–8]. This situation contributes to serious morbidity because of long diagnostic delays (mean: 2.3 to 5.2 years) [2,3,5]. Additionally, suboptimal growth monitoring leads to large numbers of futile child referrals to specialists and diagnostic workups for children with normal variants of growth, along with their related anxiety and healthcare costs [6–8]. Hence, optimizing the sensitivity and specificity of growth monitoring in primary care by a more accurate definition of abnormal growth is pivotal.

Current definitions of abnormal growth, taught during medical curricula, used in daily practice by primary care physicians, and proposed in the literature, are heterogeneous [9–11]. They vary widely in the auxological parameters used, their combinations, and the thresholds applied to define abnormal growth. For example, the World Health Organization (WHO) defines abnormal growth as standardized height < 2 standard deviations (SD) [12], whereas more complex algorithms, such as the Grote clinical decision rule, combine standardized height and body mass index, standardized growth deflection, standardized height velocity, and/or distance to standardized target height [3,13,14]. None of these algorithms has been fully evaluated according to methodological standards for clinical decision rules [15,16]. An external evaluation study of all these abnormal growth definitions has shown their low diagnostic performance in terms of sensitivity, specificity, or median theoretical reduction in time to diagnosis [17].

The poor performance of the current practices and definitions likely stems from the highly complex nature of growth monitoring. Indeed, growth monitoring aims at detecting conditions with very different patterns expressed at various ages of childhood, with variable impacts on height and weight, among a population with numerous non-pathological growth pattern variations. Complex mathematical models, now often referred to as artificial intelligence (AI), have shown their ability to handle that type of data, including in pediatrics [18]. The digitalization of medical records in primary care allows for applying AI to routine growth monitoring [11]. To date, only one AI-based algorithm has been proposed for growth monitoring [19], based on the Jenss-Bayley non-linear mixed model [20–22] which seems to offer much higher diagnostic performance than existing algorithms. However, this algorithm was designed solely to detect TS and has never been evaluated since its publication nearly 20 years ago.

Here, we aimed to develop and internally and externally evaluate an innovative and accurate AI-based approach to support frontline healthcare providers in routine pediatric practice for the early detection of abnormal height curves that require in-depth analysis and/or specialized expertise to rule out a pathological underlying process among apparently healthy children, starting with two of its main targets, GHD and TS [23].

## Population and methods

### Study design and participants

We used a case-referent approach by developing and evaluating a new algorithm from growth data of diseased children recruited in hospitals (cases) and non-diseased children from the general population (referents). The study was reported according to the updated transparent reporting of a multivariable prediction model for individual prognosis or diagnosis statement for reporting clinical prediction models that use regression or machine-learning methods (**S1 Table**) [24].

#### Development and internal evaluation.

**Fig 1:**
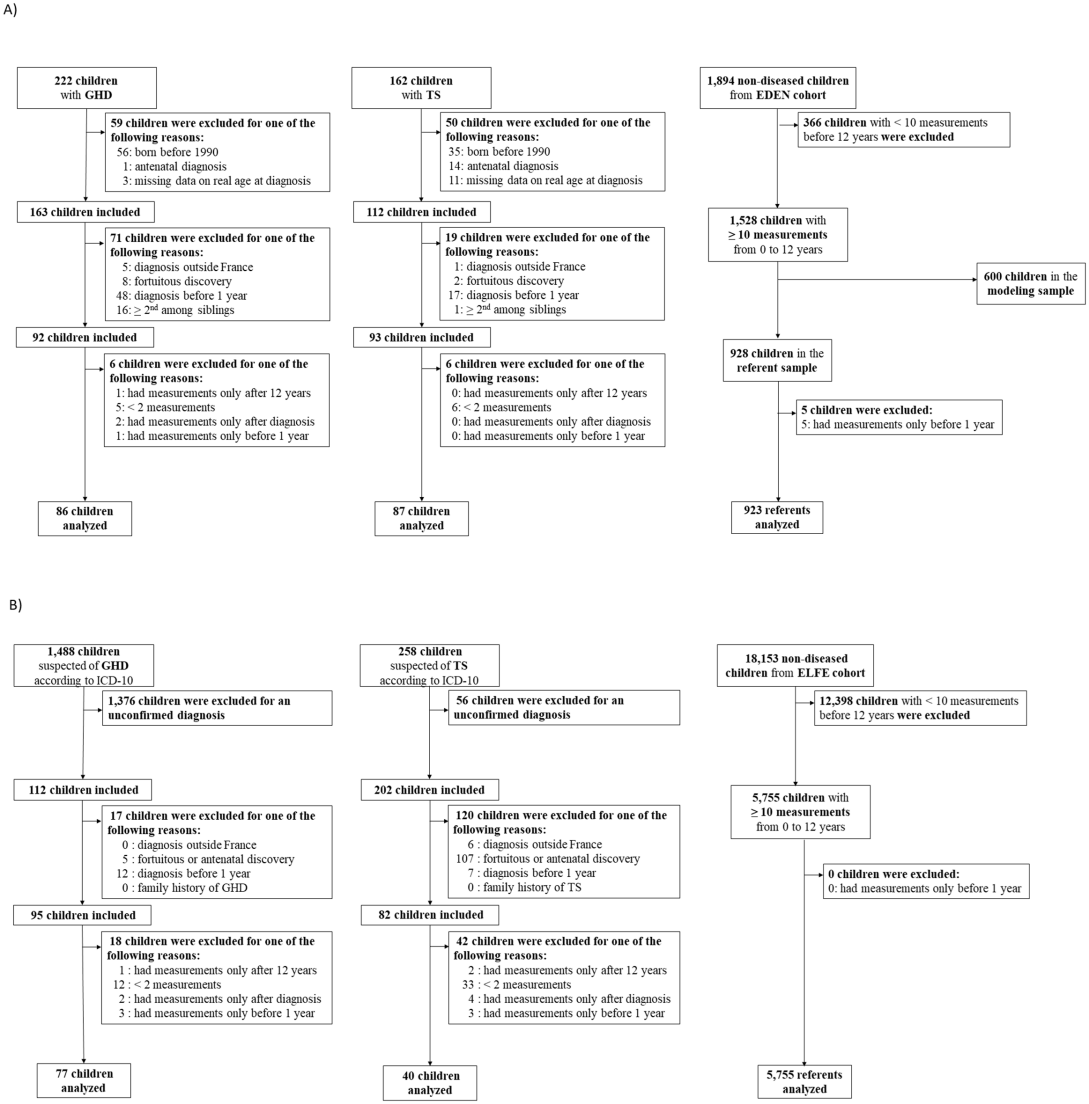
Flow charts of cases of growth hormone deficiency (GHD) or Turner syndrome (TS) and non-diseased children used as referents or for the modeling sample for development and internal evaluation (A) and external evaluation (B).

Diseased children were children with GHD associated with pituitary stalk interruption syndrome or TS, who were born between 1990 and 2014 and followed in the departments of pediatric endocrinology of Robert-Debré and Fondation Rothschild hospitals in Paris, France (**Fig 1**). Data related to these children are described in detail elsewhere [17]. Briefly, the diagnosis of GHD associated with pituitary stalk interruption syndrome was based on low insulin-like growth factor-1 level, abnormal growth hormone stimulation test results, and pituitary stalk interruption syndrome detected on MRI. TS was diagnosed by karyotyping (X0 or mosaic). We excluded cases diagnosed before age 1 year or during a fortuitous discovery (e.g., brain MRI after accidental head trauma leading to the discovery of pituitary stalk interruption syndrome), diagnosed as a second case (or more) among siblings, and cases living outside France at the date of diagnosis (because of potential variability in referral and diagnostic procedures across countries that may affect the theoretical reduction in time to diagnosis), as suggested previously [17]. We included only cases with at least two height measurements available between birth and age 12 years and with at least one measurement after age 1 year. We used only the growth data collected before the diagnosis of GHD or TS to avoid confounding by treatments such as growth hormone. This study was approved by the Necker-Enfants malades ethics committee and the National Commission for Data Protection (no. 1841989). In accordance with French law, parents and older children were informed of the use of routinely collected medical data and given the option to withdraw from the study.

Non-diseased children were apparently healthy children included in the EDEN mother–child cohort on the basis of prenatal and early postnatal determinants of child health and development (EDEN, eden.vjf.inserm.fr/). This cohort includes 1,894 children born in France between 2003 and 2006 [25]. We selected children with at least 10 height measurements collected between birth and age 12 years, and at least one measurement occurring after age 1 year. We used a random selection of non-diseased children stratified on sex to obtain a balanced subsample called “referents”. The remaining non-diseased children (n = 600) constituted the “modeling sample,” used as controls to sequentially model the height growth curve of children with the Jenss-Bayley model (see below). For referents, written informed consent was obtained from both parents at birth.

#### External evaluation.

Diseased children were identified in the Assistance Publique-Hôpitaux de Paris (AP-HP) clinical data warehouse (https://eds.aphp.fr/). This clinical data warehouse contains the electronic health records (comprehensive clinical, biological, and imaging data) of children admitted to 39 tertiary-care university hospitals in the Paris metropolitan area. Cases with GHD associated with pituitary stalk interruption syndrome or TS admitted from 2015 to 2021 were eligible (see details in **S1 Box**). We used the same inclusion and exclusion criteria as above for diseased children. The French Data Protection Agency (CNIL, Commission Nationale de l’Informatique et des Libertés) authorized the constitution of the Assistance Publique-Hôpitaux de Paris (AP-HP) clinical data warehouse on January 19, 2017 (n°19800120). In a single commitment to the CNIL, AP-HP declared complying with the French national reference methodology MR-004 governing the processing of personal data for the purposes of research of a public interest nature that does not involve the human person, in particular research reusing previously registered data. This study was approved by the Institutional Review Board of the AP-HP clinical data warehouse (IRB 00011591) (no. CSE-21–06_EBGM V on April 15, 2021).

Non-diseased children were apparently healthy children included in the *Etude Longitudinale Française depuis l’Enfance (*ELFE, https://www.elfe-france.fr/) national mother–child cohort [26]. This cohort included 18,153 children born in France in 2011. We selected children with at least 10 height measurements collected between birth and age 12 years, and with at least one measurement occurring after age 1 year.

### Data collection and auxological parameters

Demographic data (sex, gestational age), auxological data (height and weight), and data related to inclusion and exclusion criteria for cases were extracted from health records. A data-cleaning process was applied to growth data to detect and delete measurement or transcription errors (detailed in previous publications) [27–29]. Briefly, an automatic algorithm was applied, based on three stages: i) removing duplicates, and in case of two distinct values at the same age, keep the value with the z-score (according to WHO growth charts [12]) closest to the interpolated value between the previous and the next ones (if one of the two measures was not available, then the previous or next measurement was chosen); ii) deleting the absolute z-scores > 5 SD; and iii) deleting absolute z-score variations (i.e., z-score variations between two successive measurements whatever the time between the two measurements) less than the 1^st^ percentile or greater than the 99^th^ percentile of their distributions, corresponding to -0.5 SD and 1.0 SD changes for weight, -1.0 SD and 1.3 SD changes for height.

The following auxological parameters were used: standardized height and weight based on the 2018 French growth charts [28], distance to standardize target height based on the Tanner equation [30], height deflection per time interval, and standardized height deflection. These auxological parameters were required for the external evaluation study of the existing algorithms (see below) and for comparing their diagnostic performance against that of the AI algorithm [17].

### Jenss-Bayley model

The Jenss-Bayley model was developed to describe child growth curves from birth to age 12 years [20–22]. With this non-linear mixed regression model, we modeled the height growth trajectories for each child by using all their height measurements according to a maximum of five parameters (A, B, C, D, E), each with a specific interpretation in terms of growth patterns (**S2 Box**). Briefly, *A* corresponds to the height at birth, *B* the curve slope after age 2 years, *C* the spurt of growth between birth and age 2 years, and *D* the curvature of the trajectory between birth and age 2 years; *E* was added for modeling the pre-pubertal height acceleration after age 8 years.

First, as a preliminary descriptive step, we graphically described the mean height growth curve for GHD cases, TS cases, and referents, stratified by sex, using the Jenss-Bayley model. We added interaction terms between these growth parameters and clinical status (GHD cases, TS cases, and referents) to compare the mean growth parameters for GHD or TS cases with those of referents by using the Wald test [31]. We then plotted the mean modeled height growth curves as a function of age for each clinical status.

**Fig 2:**
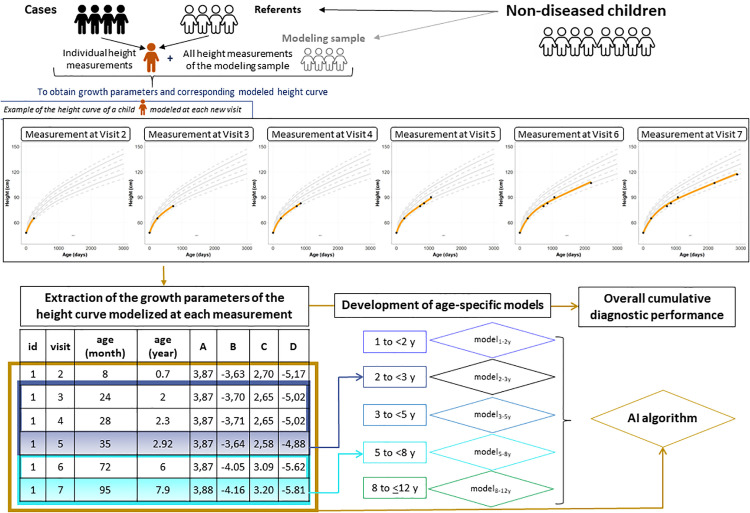
Development of the artificial intelligence (AI) algorithm. First, we modeled the height growth curve for each child (case or referent) at each new visit by pooling with all height measurements of children in the modeling sample, and therefore obtained a set of growth parameters. Second, we selected only the set of growth parameters obtained at the oldest chronological age (lines highlighted in yellow or brown) for developing and evaluating the predictive models for each age range. Once the age-specific predictive models were developed and evaluated, and the risk thresholds achieving the pre-defined specificity were identified, we assessed the cumulative diagnostic performance of the AI algorithm by applying the age-specific predictive models and corresponding risk thresholds to all the available growth measurements for children (see green line).

Second, we used the Jenss-Bayley model to model the height growth curve for each GHD case, TS case, and referent at each new visit (**Fig 2** and **S1 Fig**). To match medical practices, all available height measurements for each child at each new visit were pooled with all height measurements of children in the modeling sample. Hence, we obtained a set of growth parameters from the Jenss-Bayley model for each new visit for each child. These growth parameters were then used in the following steps to develop predictive models. The Jenss-Bayley model enables the use of growth data for all children included who had at least two height measurements, and does not impose a similar number of measurements per child. Therefore, there were no missing data to deal with. We used all available growth data for diseased and non-diseased children to maximize the precision of growth modeling and point estimates. Thus, no sample size calculation was performed.

### Predictive models

We used multinomial logistic regression modeling to predict the individual risk of each child having GHD or TS as compared with “referents” as the reference category (**S3 Box**). We pre-defined five age ranges (1 to <2, 2 to <3, 3 to <5, 5 to <8, and 8 to ≤12 years) developing a predictive model for each age range. We defined these five age ranges to capture distinct growth velocity phases and to ensure balanced distribution of measurement numbers across age ranges. In each predictive model, the predictive variables were the Jenss-Bayley growth parameters previously obtained from the Jenss-Bayley growth model (with 4 or 5 parameters depending on the age range), and the predictive model was adjusted for sex. If, for a given child and a given age range, multiple sets of the Jenss-Bayley growth parameters were available owing to multiple measurements, we selected only the one calculated at the oldest chronological age. Multicollinearity was assessed with Spearman’s pairwise correlation, the variance inflation factor (VIF), and tolerance. A correlation coefficient ≥ 0.80 and a VIF ≥ 5 (equivalent to tolerance ≤ 0.2) indicated strong multicollinearity. We found no multicollinearity between the predictive variables (**S2 Fig**) [15]. No pre-processing was applied to the growth parameters because they were on comparable scales. For each age-specific predictive model, we used the exact equation of the multinomial logistic regression as a prediction model (**Fig 2**), and we computed the risk prediction of having GHD or TS for each child. This risk prediction for each child ranged from 0 to 1 for each diagnostic category (GHD, TS, and referents).

To evaluate the diagnostic performance for each of the five age-specific predictive models, we dichotomized disease status (diseased [GHD or TS] vs. non-diseased) and risk predictions (positive algorithm vs. not) (**S3 Box**). Indeed, the dichotomization of risk prediction facilitates a clinically relevant interpretation by assessing the presence of disease, which is more interpretable in clinical settings, and enables the use of conventional diagnostic performance metrics (e.g., sensitivity and specificity) [15]. For each age-specific predictive model, we calculated the area under the receiver operating characteristic (ROC) curve (AUROC) with its 95% confidence interval (CI) and the Brier score, displaying ROC and calibration curves and decision curve analysis [32,33]. For each age-specific predictive model, we retained the risk positivity threshold offering a pre-defined specificity of >98% and calculated the corresponding sensitivity. Such a threshold limits the rate of false-positive results to 2%. By selecting risk thresholds offering 98% specificity in each of the five age ranges, we anticipated that the algorithm would have a specificity of at least of 90% which was suggested as sustainable for screening procedures in this clinical context [3,13,14]. To improve the specificity, we also studied diagnostic performance using risk positivity thresholds offering a pre-defined specificity >99% in each of the five age-specific predictive models.

Once the five age-specific predictive models were developed and the risk thresholds achieving the pre-defined specificity were identified, we assessed the cumulative diagnostic performance of the algorithm by applying the five age-specific predictive models and corresponding risk thresholds to all the available growth measurements for GHD, TS, and referents. The cumulative sensitivity of the algorithm was defined as the proportion of cases (globally or separately between GHD and TS) with at least one predicted risk over the thresholds in any of the age-specific predictive models before the actual age at diagnosis. The cumulative specificity was defined as the proportion of referents without any predicted risks over the thresholds across age-specific predictive models. The corresponding 95% CIs were calculated using the exact binomial method. We also calculated the median (interquartile range [IQR]) theoretical reductions in time to diagnosis as the difference between the actual age at diagnosis for cases and the age at which the algorithm became positive for the first time. We arbitrarily set a value of zero for the theoretical reduction in time to diagnosis for children not detected before the real age at diagnosis [17].

We evaluated the five age-specific predictive models in terms of discrimination, calibration, and cumulative diagnostic performance, both internally using five-fold cross-validation (**S3 Box**) and externally by applying the age-specific predictive models and corresponding risk thresholds to all the available growth measurements for GHD, TS, and referents.

For all analyses, we used R 4.5.2 (R Foundation for Statistical Computing, Vienna, Austria). We used the SAEMIX package (v3.4) and the CARET package (v7.0-1). The minimal data and code supporting the findings of this study are available on GitHub and archived on Zenodo [34].

### Complementary analyses

First, we compared the cumulative diagnostic performance of the algorithm with that obtained using i) the multinomial logistic regression with the synthetic minority over-sampling technique for artificially generating data from the minority outcome classes to account for unbalanced classes [35], then ii) the extreme gradient boosting regression.

Second, we compared the sensitivity, specificity, and median theoretical reductions in time to diagnosis of the algorithm and all other existing algorithms (detailed in a previous publication) [17] using the development and external evaluation datasets. For the existing algorithms with high specificity (>95%), we used the McNemar test for paired data (with a continuity correction if necessary) to compare sensitivities and specificities.

## Results

### Population and growth data

#### Development and internal evaluation.

Among the potentially eligible 222 cases with GHD, 162 with TS, and 928 referents, applying the exclusion criteria led to a study sample of 86 cases of GHD, 87 cases of TS, and 923 referents (**Fig 1**). Mean age at diagnosis was 5.73 (95% CI 5.00-6.46) and 9.03 (95% CI 8.23-9.84) years for children with GHD and TS, respectively. Before diagnosis, cases of GHD or TS had a median number of measurements per child of 8 (IQR 5–11) and 9 (6–11), respectively. Referents had a median number of measurements per child of 21 (16–26), including 17 (14–20) before age 49 months and 20 (16–25) before age 109 months (**S2 Table**).

**Fig 3:**
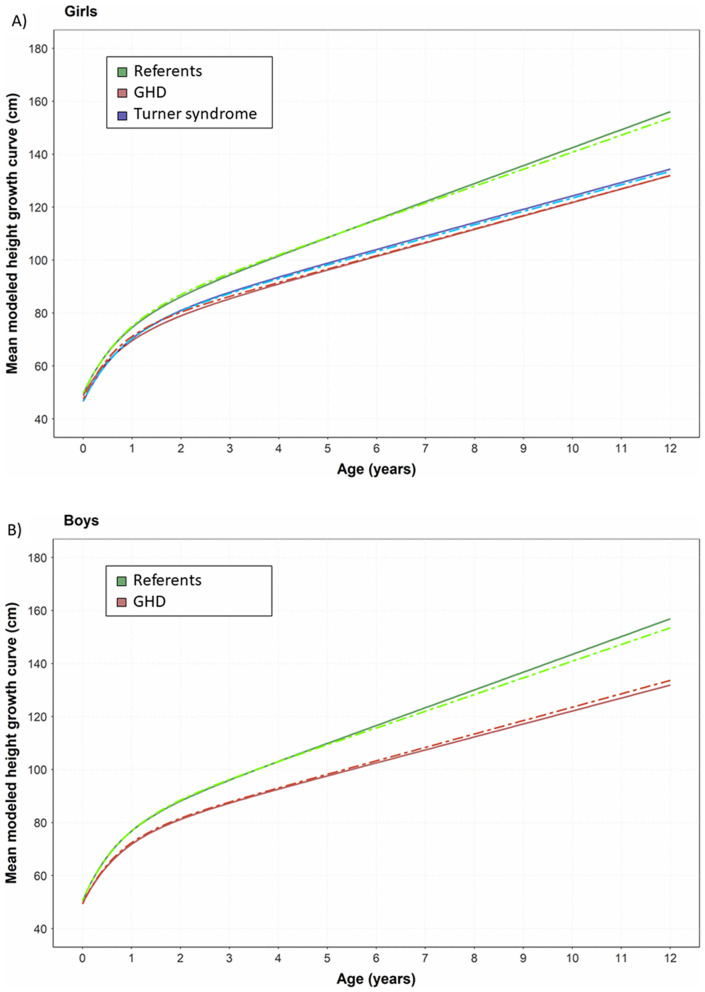
Mean modeled individual height growth curves for growth hormone deficiency (GHD) cases, Turner syndrome cases, and referents from birth to age 12 years for girls (A) and boys (B). (development ―; external evaluation ---).

The mean height growth curves for GHD or TS cases differed graphically from those for referents (**Fig 3**), with statistically significant differences for the A, B, and C parameters of the Jenss-Bayley model (**Table 1** and **S3 Fig**). The D parameter differed significantly between TS cases and referents. The E parameter did not significantly differ between cases and referents.

**Table 1:** Development: mean (and standard error) of the five growth parameters from the Jenss-Bayley model for growth hormone deficiency (GHD) cases, Turner syndrome (TS) cases, and referents from birth to age 12 years.

	GHD (n = 86)		TS (n = 87)	Referents (n = 923)	
Mean (standard error)	Girls	Boys	Girls	Girls	Boys
A: height at birth	3.89 (0.009)^*^	3.90 (0.007)^*^	3.84 (0.005)^*^	3.91 (0.002)	3.92 (0.002)
B: curve slope > 2 years	−4.28 (0.100)^*^	−4.33 (0.067)^*^	−4.30 (0.043)^*^	−3.98 (0.010)	−4.00 (0.009)
C: spurt of growth from 0 to 2 years	3.08 (0.066)^*^	3.14 (0.041)^*^	3.29 (0.030)^*^	3.19 (0.009)	3.22 (0.008)
D: curvature of the trajectory from 0 to 2 years	−5.64 (0.096)	−5.56 (0.062)	−5.71 (0.049)^*^	−5.58 (0.015)	−5.49 (0.013)
E: pre-pubertal height acceleration > 8 years	−19.85 (120.51)	−19.34 (49.11)	−19.38 (27.90)	−19.45 (8.58)	−19.45 (8.89)
Number of children	28	58	87	461	462
Number of measurements	247	460	758	9,826	9,814

* Significant difference in growth parameters of GHD or TS cases as compared with referents of the corresponding sex (p for interaction < 0.05).

#### External evaluation.

Among the potentially eligible 112 cases of GHD, 202 cases of TS, and 5,755 referents, applying the exclusion criteria led to a study sample of 77 cases of GHD, 40 cases of TS, and 5,755 referents (**Fig 1**). Mean age at diagnosis was 4.91 (95% CI 4.79-5.04) and 7.89 (95% CI 6.69-9.09) years for children with GHD and TS, respectively. The median number of measurements per child in GHD cases, TS cases, or referents was 6 (4–10), 10 (7–12), and 11 (10–12), respectively (**S3 Table**).

The mean height growth curves for GHD or TS cases differed graphically from those for referents (**Fig 3**), with statistically significant differences for the A and B parameters of the Jenss-Bayley model (**Table 2**). The C parameter differed significantly between GHD cases and referents. The D parameter differed significantly between girl GHD cases and referents. The E parameter did not significantly differ between cases and referents.

**Table 2:** External evaluation: mean (and standard error) of the five growth parameters from the Jenss-Bayley model for growth hormone deficiency (GHD) cases, Turner syndrome (TS) cases, and referents from birth to age 12 years.

	GHD (n = 77)		TS (n = 40)	Referents (n = 5,755)	
Mean (standard error)	Girls	Boys	Girls	Girls	Boys
A: height at birth	3.86 (0.008)^*^	3.89 (0.007)^*^	3.84 (0.007)^*^	3.91 (0.001)	3.93 (0.007)
B: curve slope > 2 years	−4.31 (0.089)^*^	−4.29 (0.086)^*^	−4.30 (0.075)^*^	−4.04 (0.006)	−4.06 (0.006)
C: spurt of growth from 0 to 2 years	3.10 (0.052)^*^	3.15 (0.048)^*^	3.26 (0.046)	3.27 (0.005)	3.30 (0.004)
D: curvature of the trajectory from 0 to 2 years	−5.47 (0.080)^*^	−5.53 (0.066)	−5.71 (0.063)	−5.70 (0.006)	−5.62 (0.006)
E: pre-pubertal height acceleration > 8 years	−19.17 (57.43)	−19.38 (72.79)	−19.39 (55.09)	−19.49 (5.94)	−19.45 (5.47)
Number of children	28	49	40	2,851	2,904
Number of measurements	204	372	371	32,318	32,175

* Significant difference in growth parameters of GHD or TS cases as compared with referents of the corresponding sex (p for interaction < 0.05).

### Development and internal and external evaluations of the AI algorithm

**Fig 4:**
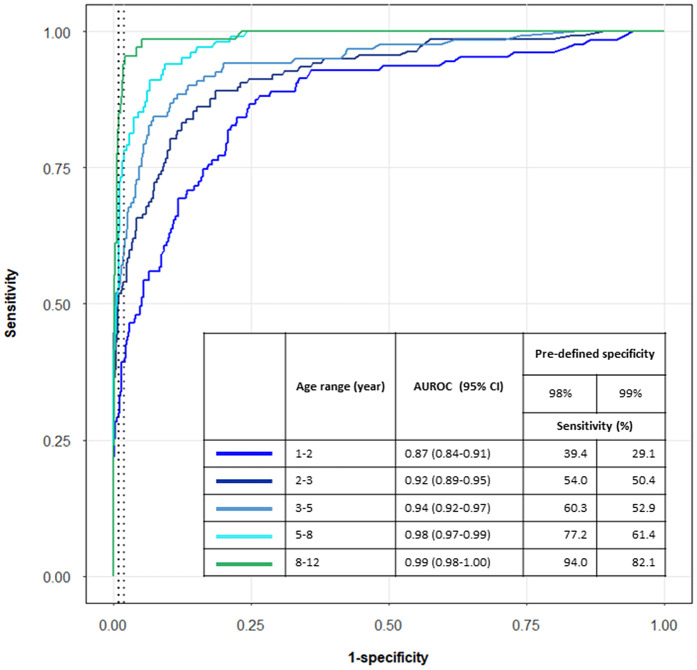
Development: area under the receiver operating characteristic curves (AUROCs) for age-specific predictive models. CI: confidence interval.

**Fig 5:**
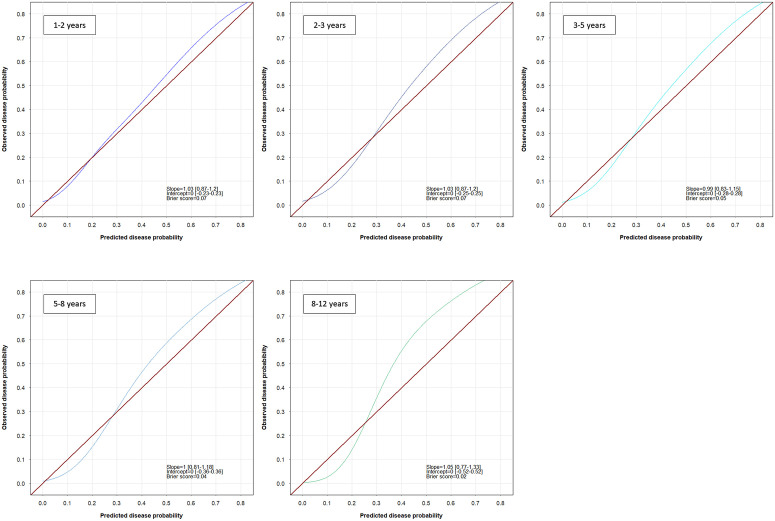
Development: calibration curves for age-specific predictive models.

**Fig 6:**
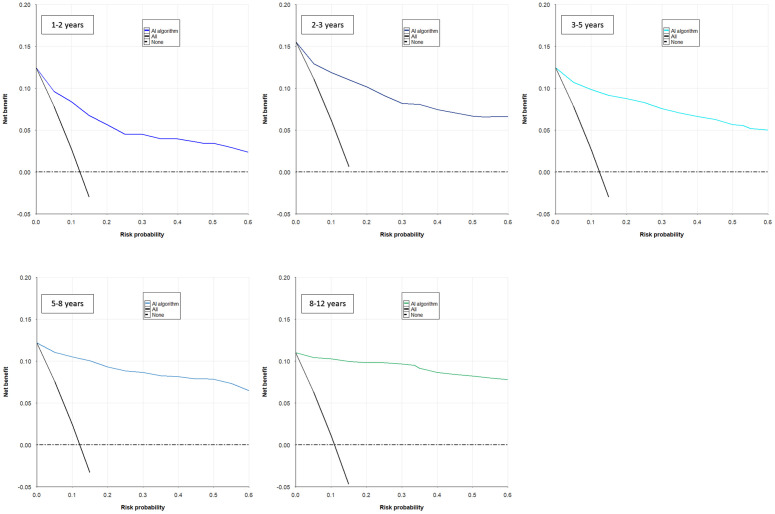
Development: decision curve analysis for age-specific predictive models.

For each age-specific predictive model, AUROCs ranged from 0.87 to 0.99 (**Fig 4**). With the identified risk thresholds offering the pre-defined specificities >98% and >99%, the corresponding sensitivities ranged from 39.4% to 94.0% and 29.1% to 82.1% at age 1–2 and 8–12 years, respectively. The calibration curves showed high consistencies between predicted and observed risks, notably for the highest predicted probabilities (**Fig 5**), and the Brier scores indicated satisfactory diagnostic performance of the predicted risks. On decision curve analysis, the predictive models provided a high net benefit across risk probabilities (Fig 6). The calculated coefficients and thresholds retained to obtain the pre-defined specificities are in the supplemental materials (**S4 Table**).

With the identified risk thresholds offering pre-defined specificity >98% for the five age-specific equations, the algorithm had a cumulative sensitivity of 89.6% (95% CI 84.1-93.7), 88.4% (79.7-94.3) for GHD cases and 90.8% (82.7-95.9) for TS cases, with a cumulative specificity of 94.7% (93.0-96.0) (**Table 3 and Table 4**). The median (IQR) theoretical reduction in time to diagnosis was 2.8 (1.3-5.3) years: 1.8 (0.7-3.5) for GHD cases and 4.2 (2.1-7.0) for TS cases. With the identified risk thresholds offering pre-defined specificities >99% for the five-age range-specific equations, the algorithm had a cumulative sensitivity of 82.7% (95% CI 76.2-88.0): 81.4% (71.6-89.0) for GHD cases and 83.9% (74.5-90.9) for TS cases, and a cumulative specificity of 97.4% (96.2-98.3) (**Tables 3 and 4**). The median (IQR) theoretical reduction in time to diagnosis was 2.6 (1.1-5.0) years, 1.8 (0.8-3.4) for GHD cases and 3.6 (1.2-5.8) for TS cases.

**Table 3:** Development: cumulative diagnostic performance of the artificial intelligence algorithm for predicting the individual risk of cases (growth hormone deficiency or Turner syndrome).

	Pre-defined specificity								
	>98%					>99%			
Cumulative diagnostic performance by age category (y)	Sensitivity (N = 173)		Specificity (N = 923)			Sensitivity (N = 173)		Specificity (N = 923)	
	*%* *(n cases)*	*95% CI*	*%* *(n referents)*	*95% CI*		*%* *(n cases)*	*95% CI*	*%* *(n referents)*	*95% CI*
1 to <2	28.9 (50)	22.3–36.3	97.2 (897)	95.9–98.2		22.5 (39)	16.5–29.5	98.7 (911)	97.7–99.3
1 to <3	49.7 (86)	42.0–57.4	96.6 (892)	95.3–97.7		45.7 (79)	38.1–53.4	98.3 (907)	97.2–99.0
1 to <5	62.4 (108)	54.8–69.7	95.7 (883)	94.1–96.9		56.1 (97)	48.3–63.6	97.9 (904)	96.8–98.8
1 to <8	78.0 (135)	71.1–84.0	95.0 (877)	93.4–96.3		70.5 (122)	63.1–77.2	97.5 (900)	96.3–98.4
1 to ≤12	89.6 (155)	84.1–93.7	94.7 (874)	93.0–96.0		82.7 (143)	76.2–88.0	97.4 (899)	96.2–98.3
**Theoretical reduction in time to diagnosis (y),** median (IQR)	2.8	1.3–5.3				2.6	1.1–5.0		

CI: confidence interval, IQR: interquartile range.

**Table 4:** Development: cumulative diagnostic performance of the artificial intelligence algorithm by condition.

	Pre-defined (and calculated) specificity*							
	>98% (94.7%)				>99% (97.4%)			
	GHD (N = 86)		TS (N = 87)		GHD (N = 86)		TS (N = 87)	
	*% (n)*	*95% CI*	*% (n)*	*95% CI*	*% (n)*	*95% CI*	*% (n)*	*95% CI*
**Sensitivities** (age 1–12 years)	88.4 (76)	79.7–94.3	90.8 (79)	82.7–95.9	81.4 (70)	71.6–89.0	83.9 (73)	74.5–90.9
**Theoretical reduction in time to diagnosis (y)**, median (IQR)	1.8	0.7–3.5	4.2	2.1–7.0	1.8	0.8–3.4	3.6	1.2–5.8

CI: confidence interval, IQR: interquartile range, GHD: growth hormone deficiency, TS: Turner syndrome.

* The calculated specificity corresponds to the cumulative specificities calculated from referents from age 1–12 years during the development (see Table 3).

**Fig 7:**
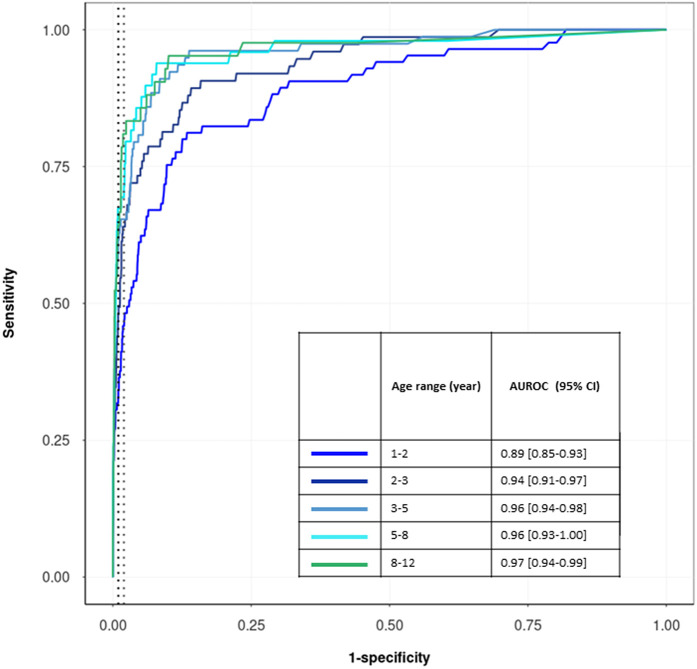
External evaluation: area under the receiver operating characteristic curves (AUROCs) for age-specific predictive models. CI: confidence interval.

**Fig 8:**
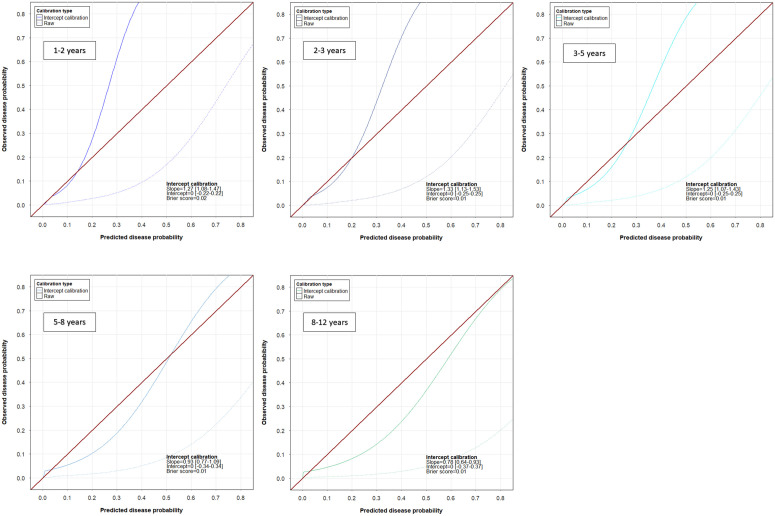
External evaluation: calibration curves for age-specific predictive models.

**Fig 9:**
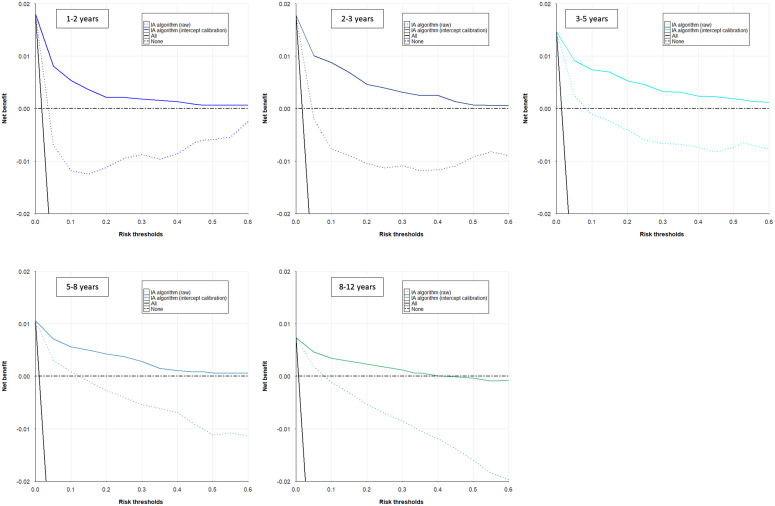
External evaluation: decision curve analysis for age-specific predictive models.

Internal and external evaluations showed stable results in terms of discrimination (**S4 Fig** and **Fig 7**), calibration (**S5 Fig** and **Fig 8**), or decision curve analysis (**S6 Fig** and **Fig 9**) for each of the five age-specific predictive models. The cumulative diagnostic performance from internal and external evaluations is in **S5** and **S6 Tables** and **Table 5** and **Table 6**, respectively. With the identified risk thresholds offering pre-defined specificity >98% for the five age-specific equations, the external evaluation study revealed a cumulative sensitivity of 84.6% (95% CI 76.8-90.6), 81.8% (71.4-89.7) for GHD cases and 90.0% (76.3-97.2) for TS cases, with a cumulative specificity of 94.3% (93.6-94.9). The median (IQR) theoretical reduction in time to diagnosis was 2.0 (0.6-3.8) years, 1.6 (0.5-2.8) for GHD cases and 3.0 (1.0-5.4) for TS cases.

**Table 5:** External evaluation: cumulative diagnostic performance of the artificial intelligence algorithm for predicting the individual risk of cases (growth hormone deficiency or Turner syndrome).

Pre-defined specificity	98%				99%			
Cumulative diagnostic performance	Sensitivity (N = 117)		Specificity (N = 5,755)		Sensitivity (N = 117)		Specificity (N = 5,755)	
	*%* *(n cases)*	*95% CI*	*%* *(n referents)*	*95% CI*	*%* *(n cases)*	*95% CI*	*%* *(n referents)*	*95% CI*
1–2 years	30.8 (36)	22.6–40.0	98.7 (5,679)	98.3–99.0	23.1 (27)	15.8–31.8	99.6 (5,730)	99.4–99.7
1–3 years	53.0 (62)	43.5–62.3	97.9 (5,636)	97.5–98.3	43.6 (51)	34.4–53.1	99.0 (5,695)	98.7–99.2
1–5 years	69.2 (81)	60.0–77.4	97.2 (5,593)	96.7–97.6	61.5 (72)	52.1–70.4	98.5 (5,668)	98.1–98.8
1–8 years	75.2 (88)	66.4–82.7	96.5 (5,553)	96.0–97.0	66.7 (78)	57.4–75.1	98.2 (5,653)	97.9–98.6
1–12 years	84.6 (99)	76.8–90.6	94.3 (5,425)	93.6–94.9	76.9 (90)	68.2–84.2	96.6 (5,561)	96.1–97.1
**Theoretical reductions in time to diagnosis (y),** median [IQR]	2.0	0.6–3.8			1.8	0.6–3.6		

CI: confidence interval, IQR: interquartile range.

**Table 6:** External evaluation: cumulative diagnostic performance of the artificial intelligence algorithm by condition.

	Pre-defined (and calculated) specificity*							
	>98% (94.3%)				>99% (96.6%)			
	GHD (N = 77)		TS (N = 40)		GHD (N = 77)		TS (N = 40)	
	*% (n)*	*95% CI*	*% (n)*	*95% CI*	*% (n)*	*95% CI*	*% (n)*	*95% CI*
**Sensitivities** (age 1–12 years)	81.8 (63)	71.4–89.7	90.0 (36)	76.3–97.2	72.7 (56)	61.4–82.3	85.0 (34)	70.2–94.3
**Theoretical reduction in time to diagnosis (y)**, median (IQR)	1.6	0.5–2.8	3.0	1.0–5.4	1.6	0.5–2.8	2.6	0.8–4.8

CI: confidence interval, IQR: interquartile range, GHD: growth hormone deficiency, TS: Turner syndrome.

* The calculated specificity corresponds to the cumulative specificities calculated from referents from age 1–12 years during the external evaluation (see Table 5).

### Complementary analyses

During the development and evaluation, the diagnostic performance of multinomial logistic regression with the synthetic minority over-sampling technique and the extreme gradient boosting regression was similar to that of multinomial logistic regression (**S7 Table**).

The comparison of the diagnostic performance between the algorithm and existing algorithms is in **S8 Table**. With the development dataset, the specificity of the existing algorithms ranged from 25.9% to 90.9%. Only the revised Coventry consensus had a specificity > 90% (i.e., 90.9%) with a sensitivity of 79.2% and a median theoretical reduction in time to diagnosis of 2.1 years. The algorithm had significantly higher sensitivity (89.6%), specificity (94.7%), and median theoretical reduction in time to diagnosis of 2.8 years. With the external evaluation dataset, the sensitivities of the algorithm and the revised Coventry consensus did not statistically significantly differ (84.6% vs. 81.2%), and its specificity was significantly lower (94.3% vs. 95.7%), with a close median theoretical reduction in time to diagnosis (2.0 vs. 1.2 years).

## Discussion

### Main findings and interpretation

In this case-referent study, we developed and internally and externally evaluated an AI algorithm for growth monitoring to predict the individual risk of GHD or TS in a child from age 1–12 years. The five age-specific predictive models achieved good discrimination and calibration. The algorithm had high cumulative diagnostic performance during the development, with sensitivities for GHD or TS cases ranging from 82.7% to 89.6% and specificities ranging from 94.7% to 97.4%. The slightly higher sensitivities for TS were previously reported [17] and may be related to a more homogeneous growth phenotype in TS vs. GHD. Notably, some GHD cases may exhibit near-normal height growth even in the presence of pituitary stalk interruption syndrome [36].

The algorithm was based on classical approaches combining the Jenss-Bayley non-linear mixed growth model and multinomial logistic regression. The use of a two-stage approach, incorporating the minimal set of growth parameters from the Jenss-Bayley model into predictive models, allowed for considering the non-linear association between height and age as well as the repeated measurements. As frequently emphasized in the literature [37], an alternative AI approach (i.e., extreme gradient boosting) did not enhance predictive abilities.

Of note, although many AI-based predictive models lack external evaluation or fail during it [24], the algorithm achieved cumulative sensitivities ranging from 76.9% to 84.6% and specificities from 94.3% to 96.6% during a temporal and geographic external evaluation, among large regional and national samples of diseased and non-diseased children. This result confirms the temporal stability of the algorithm despite an evolution of clinical and therapeutic practices during the long study period.

The AI algorithm had better diagnostic performance than existing ones. Only the revised Coventry consensus had slightly lower or similar performance as compared with the AI algorithm, with sensitivity 79.2% (vs. 89.6%), specificity 90.9% (vs. 94.7%), and a median theoretical reduction in time to diagnosis 2.1 years (vs. 2.8 years). The improvement in diagnosis performance observed with the AI algorithm is probably related to the use of almost all the information about the growth of each child, the use of the most appropriate growth model proposed by Jenss-Bayley, and multiclass prediction modeling.

Our approach can be considered a refinement of the screening rule developed in 2005 by van Dommelen *et al*. [19]. The authors combined the Jenss-Bayley non-linear mixed model and discriminant analysis to predict the individual risk of each child having TS from birth to age 8. This screening rule had a sensitivity of 90.7% and a specificity of 98.0% during the development step, similar to our algorithm. However, since the development of this screening rule, no internal or external evaluation studies have been performed, and no extension has been proposed for predicting other conditions, such as GHD.

### Limitations

First, although we aimed to develop and internally and externally evaluate an algorithm that could be used in all countries with advanced economies, the use of cases from various Paris-area hospitals and referents from two French cities and a population-based national study might have introduced a potential selection bias, which may have affected the diagnostic performance of the algorithm and limits its generalizability to other populations. Additionally, the relatively limited number of cases used to develop the five age-specific predictive models may have led to low power and overfitting. However, the internal and external evaluations revealed only a slight reduction in diagnostic performance and thus their robustness.

Second, we did not have access to further information on cases, such as socioeconomic status or ethnicity, which prevented us from comparing children included in the development and external evaluation stages.

Third, the measurements were collected from routine practice without standardization and often with limited numbers of measures, which could have introduced bias or non-systematic errors and affected the reproducibility of predictive models. However, the robustness of the diagnostic performance during external evaluation suggests that any such bias or errors are likely minimal.

Fourth, the algorithm was built using height data for GHD cases defined with strict diagnostic criteria, as suggested in a previous consensus process [23]. The stability of the diagnostic performance of the algorithm must be evaluated with data for GHD cases defined with less stringent criteria.

Fifth, we did not include in the predictive model growth parameters other than height, such as distance to the standardized target height, because parental height was not systematically collected. Van Dommelen *et al.* have shown an improvement in diagnostic performance by adding parental height and gestational age to the Jenss-Bayley growth parameters [19]. Including these parameters, as well as other clinical data, will probably improve this new algorithm too. Furthermore, the revised Coventry consensus, which includes the use of distance to the standardized target height in addition to standardized height, had slightly lower or similar performance as compared with our algorithm. Refining the algorithm by incorporating distance to the standardized target height may improve its performance.

Sixth, we restricted the target age for which the algorithm is applicable to age ≥ 1 year because diagnoses before this age are more often based on clinical signs other than growth, such as hypoglycaemia or midline defects in GHD and lymphedema or cardiac malformations in TS. Additionally, non-pathological height variations are more common among younger referents, which could affect the algorithm’s specificity.

Seventh, the algorithm was developed and internally evaluated with a specific training dataset, and its calibration is inherently dependent on these data, which may restrict its transportability to other populations.

Finally, the growth modeling and predictive models should be extended to other priority target conditions that affect growth with growth patterns other than GHD and TS. For example, celiac and Crohn’s diseases usually affect weight before and/or to a more significant extent than height [23]. The Jenss-Bayley mixed model for the weight growth curve will probably be more accurate for these target diseases in a future refined algorithm [20,21]. A joint modeling of height and weight may also be useful for target conditions that affect both weight and height [38,39] such as craniopharyngioma and hypothalamic-optochiasmatic astrocytoma.

### Usability of the AI algorithm in clinical practice

In the present study, the algorithm was developed and internally and externally evaluated as a proof of concept to demonstrate the accuracy of such an approach for the early detection of GHD and TS. Early detection of these two main target conditions is essential for the timely implementation of treatments aimed at reducing morbidities, improving psychosocial well-being and quality of life, and reducing healthcare costs. Before any implementation in daily practice, the algorithm needs to take into consideration the limitations described above. Furthermore, the algorithm will require external evaluation with data from independent populations from other countries by calibrating predictive models to these populations and adjusting the false-positive rate aligned with the capacities of the national healthcare system. Finally, although the algorithm was developed by using R free software, deployment would require embedding in a more user-friendly interface. The algorithm may be integrated into the existing medical software that supports the electronic medical records used by primary care physicians to track and monitor child growth. In such a scenario, individual height curves will be modeled using the Jenss-Bayley model (which will require the use of a control population) and interpreted by the algorithm. In case of abnormal growth trajectories, a pop-up alert could appear, indicate the most likely target conditions, and suggest the need for attention by the primary care physicians for a potential referral to a specialist. Any implementation in medical software used by primary-care healthcare practitioners or parental smartphone applications [40] will require the scrutiny of potentially unexpected side effects, such as the replacement or loss of clinical judgment, unnecessary anxiety, and futile child referrals to specialists and diagnostic workups or, in contrast, delayed diagnosis related to reassuring false negative results.

## Conclusion

We report the development and internal and external evaluations of an AI algorithm with high diagnostic performance for the early detection of GHD and TS, promising better results than with usual care or other existing algorithms. Before the algorithm can be implemented in daily practice, the transportability of its diagnostic performance should be further assessed in children with other targeted growth monitoring conditions and/or in other countries as an extension of growth data.

## Supporting information

S1 Table Transparent Reporting of a multivariable prediction model for Individual Prognosis Or Diagnosis – Artificial Intelligence statement (TRIPOD-AI). (DOCX)

S2 Table Development: number of cases and referents and available height measurements (before diagnosis for cases), by sex and age interval (in years), until age 12 years. (DOCX)

S3 Table External evaluation: number of cases and referents and available height measurements (before diagnosis for cases), by sex and age interval (in years), until age 12 years. (DOCX)

S4 Table Development: model coefficients and threshold of the artificial intelligence algorithm for predicting the individual risk of cases (growth hormone deficiency or Turner syndrome). (DOCX)

S5 Table Internal evaluation: cumulative diagnostic performance of the artificial intelligence algorithm for predicting the individual risk of cases (growth hormone deficiency or Turner syndrome). (DOCX)

S6 Table Internal evaluation: cumulative diagnostic performance of the artificial intelligence algorithm by condition. (DOCX)

S7 Table Comparison of the artificial intelligence algorithm with the multinomial logistic regression without and with Synthetic Minority Over-sampling TEchnique (SMOTE) and the extreme gradient boosting (XGBoost) regression. (DOCX)

S8 Table Comparison of the artificial intelligence algorithm with other existing algorithms. (DOCX)

S1 Fig Running the Jenss-Bayley model for sequentially modeling the height growth curve of a child and application of an artificial intelligence algorithm. (DOCX)

S2 Fig Development: multicollinearity maps. (DOCX)

S3 Fig Development: distribution of the five growth parameters, from the Jenss-Bayley model, for growth hormone deficiency (GHD) cases, Turner syndrome cases, and referents from birth to age 12 years, for girls and boys. (DOCX)

S4 Fig Internal evaluation: area under the receiver operating characteristic curves (AUROCs) for age-specific predictive models. (DOCX)

S5 Fig Internal evaluation: calibration curves for age-specific predictive models. (DOCX)

S6 Fig Internal evaluation: decision curve analysis for age-specific predictive models. (DOCX)

S1 Box Identification of cases and data extraction from the clinical data warehouse. (DOCX)

S2 Box Mixed-effect Jenss-Bayley model. (DOCX)

S3 Box Age-range specific predictive models: development and evaluation. (DOCX)
