# Supplementary material for: Development, internal and external evaluation of an artificial intelligence algorithm for child growth monitoring in primary care
Source: PLOS Digit Health. 2026 Jul 15;5(7):e0001526. doi: 10.1371/journal.pdig.0001526 (PMC13372244; doi:10.1371/journal.pdig.0001526)
Supplement: S2 Table — (DOCX) [file pdig.0001526.s002.docx]

**S2 Table.** Development: number of cases and referents and available height measurements (before diagnosis for cases), by sex and age interval (in years), until age 12 years.

|  | **GHD** (n=86) | | | | |  | **TS** (n=87) | |  | **Referents** (n=923) | | | | |
| --- | --- | --- | --- | --- | --- | --- | --- | --- | --- | --- | --- | --- | --- | --- |
|  | ***Girls*** | |  | ***Boys*** | |  | ***Girls*** | |  | ***Girls*** | |  | ***Boys*** | |
| **Number of:** | Children  (n) | Measures  (n) |  | Children  (n) | Measures  (n) |  | Children  (n) | Measures  (n) |  | Children  (n) | Measures  (n) |  | Children  (n) | Measures  (n) |
| **Age interval, y** |  |  |  |  |  |  |  |  |  |  |  |  |  |  |
| 0 to <1 | 28 | 93 |  | 55 | 135 |  | 85 | 191 |  | 461 | 4,389 |  | 462 | 4,395 |
| 1 to <2 | 22 | 63 |  | 47 | 85 |  | 60 | 88 |  | 446 | 1,684 |  | 447 | 1,601 |
| 2 to <3 | 21 | 32 |  | 45 | 65 |  | 72 | 101 |  | 373 | 778 |  | 373 | 776 |
| 3 to <4 | 14 | 20 |  | 33 | 45 |  | 52 | 67 |  | 412 | 912 |  | 418 | 893 |
| 4 to <5 | 11 | 14 |  | 21 | 29 |  | 42 | 56 |  | 233 | 305 |  | 256 | 346 |
| 5 to <6 | 5 | 7 |  | 23 | 31 |  | 46 | 55 |  | 347 | 623 |  | 364 | 680 |
| 6 to <7 | 4 | 5 |  | 17 | 20 |  | 43 | 53 |  | 189 | 214 |  | 193 | 219 |
| 7 to <8 | 3 | 4 |  | 11 | 15 |  | 32 | 37 |  | 205 | 307 |  | 200 | 302 |
| 8 to <9 | 3 | 3 |  | 7 | 11 |  | 31 | 35 |  | 150 | 176 |  | 165 | 199 |
| 9 to <10 | 3 | 4 |  | 9 | 10 |  | 25 | 32 |  | 45 | 49 |  | 44 | 46 |
| 10 to <11 | 2 | 2 |  | 6 | 7 |  | 15 | 17 |  | 115 | 141 |  | 111 | 128 |
| 11 to <12 | 0 | 0 |  | 4 | 7 |  | 22 | 26 |  | 148 | 248 |  | 151 | 228 |
| **Total** | **28** | **247** |  | **58** | **460** |  | **87** | **758** |  | **461** | **9,826** |  | **462** | **9,814** |

*GHD: growth hormone deficiency, TS. Turner syndrome*
