# Supplementary material for: Development, internal and external evaluation of an artificial intelligence algorithm for child growth monitoring in primary care
Source: PLOS Digit Health. 2026 Jul 15;5(7):e0001526. doi: 10.1371/journal.pdig.0001526 (PMC13372244; doi:10.1371/journal.pdig.0001526)
Supplement: S3 Table — (DOCX) [file pdig.0001526.s003.docx]

**S3 Table.** External evaluation: number of cases and referents and available height measurements (before diagnosis for cases), by sex and age interval (in years), until age 12 years.

|  | **GHD** (n=77) | | | | |  | **TS** (n=40) | |  | **Referents** (n=5,755) | | | | |
| --- | --- | --- | --- | --- | --- | --- | --- | --- | --- | --- | --- | --- | --- | --- |
|  | ***Girls*** | |  | ***Boys*** | |  | ***Girls*** | |  | ***Girls*** | |  | ***Boys*** | |
| **Number of:** | Children  (n) | Measures  (n) |  | Children  (n) | Measures  (n) |  | Children  (n) | Measures  (n) |  | Children  (n) | Measures  (n) |  | Children  (n) | Measures  (n) |
| **Age interval, y** |  |  |  |  |  |  |  |  |  |  |  |  |  |  |
| 0 to <1 | 25 | 75 |  | 43 | 132 |  | 36 | 109 |  | 2,851 | 12,739 |  | 2,904 | 13,068 |
| 1 to <2 | 26 | 40 |  | 36 | 72 |  | 29 | 59 |  | 2,306 | 2,774 |  | 2,331 | 2,788 |
| 2 to <3 | 16 | 22 |  | 29 | 45 |  | 30 | 44 |  | 2,074 | 2,407 |  | 2,079 | 2,402 |
| 3 to <4 | 13 | 14 |  | 27 | 42 |  | 25 | 35 |  | 1,988 | 2,436 |  | 1,994 | 2,437 |
| 4 to <5 | 9 | 16 |  | 21 | 27 |  | 19 | 25 |  | 1,754 | 2,078 |  | 1,732 | 2,058 |
| 5 to <6 | 3 | 5 |  | 15 | 18 |  | 21 | 21 |  | 1,502 | 1,517 |  | 1,579 | 1,592 |
| 6 to <7 | 4 | 5 |  | 7 | 8 |  | 13 | 15 |  | 20 | 20 |  | 22 | 22 |
| 7 to <8 | 4 | 5 |  | 6 | 6 |  | 13 | 13 |  | 1,507 | 1,515 |  | 1,434 | 1,437 |
| 8 to <9 | 6 | 8 |  | 8 | 11 |  | 14 | 17 |  | 1,504 | 1,525 |  | 1,448 | 1,474 |
| 9 to <10 | 2 | 2 |  | 5 | 5 |  | 12 | 16 |  | 523 | 532 |  | 535 | 547 |
| 10 to <11 | 4 | 8 |  | 1 | 2 |  | 6 | 9 |  | 2,674 | 4,550 |  | 2,765 | 4,683 |
| 11 to <12 | 3 | 4 |  | 3 | 4 |  | 5 | 8 |  | 208 | 225 |  | 195 | 207 |
| **Total** | **28** | **204** |  | **49** | **372** |  | **40** | **371** |  | **2,851** | **32,318** |  | **2,904** | **32,715** |

*GHD: growth hormone deficiency, TS. Turner syndrome*
