# Supplementary material for: Development, internal and external evaluation of an artificial intelligence algorithm for child growth monitoring in primary care
Source: PLOS Digit Health. 2026 Jul 15;5(7):e0001526. doi: 10.1371/journal.pdig.0001526 (PMC13372244; doi:10.1371/journal.pdig.0001526)
Supplement: S4 Table — (DOCX) [file pdig.0001526.s004.docx]

## S4 Table. Development: model coefficients and threshold of the artificial intelligence algorithm for predicting the individual risk of cases (growth hormone deficiency or Turner syndrome).

| **Model coefficients  by age category (y)** | **Intercept** | | **A** | | **B** | | **C** | | **D** | | **E** | | **Sex** | |  | **Thresholds for dichotomization of risk prediction** | |
| --- | --- | --- | --- | --- | --- | --- | --- | --- | --- | --- | --- | --- | --- | --- | --- | --- | --- |
|  | **GHD** | **TS** | **GHD** | **TS** | **GHD** | **TS** | **GHD** | **TS** | **GHD** | **TS** | **GHD** | **TS** | **GHD** | **TS** |  | 98% | 99% |
| 1 to <2 | 19.8 | 49.5 | -12.7 | -25.5 | -14.8 | -8.2 | -9.5 | -6.7 | -0.5 | 0.4 | - | - | -1.9 | 18.9 |  | 0.47 | 0.62 |
| 2 to <3 | -7.1 | 40.7 | -10.0 | -24.7 | -20.6 | -13.5 | -9.9 | -6.7 | 0.3 | 1.7 | - | - | -1.8 | 15.5 |  | 0.57 | 0.67 |
| 3 to <5 | -33.9 | 23.2 | -6.8 | -26.8 | -24.9 | -17.6 | -10.5 | -8.8 | 1.1 | 1.5 | - | - | -1.2 | 22.4 |  | 0.54 | 0.71 |
| 5 to <8 | -42.8 | 2.4 | -10.4 | -27.0 | -30.1 | -29.9 | -11.7 | -9.7 | 0.3 | 3.1 | - | - | -2.3 | 13.5 |  | 0.55 | 0.77 |
| 8 to <12 | -9.4 | -9.3 | 6.4 | -18.11 | -43.6 | -42.5 | -13.3 | -6.9 | 2.3 | 4.1 | 7.3 | 3.6 | -1.1 | 8.9 |  | 0.43 | 0.83 |

The model coefficients and thresholds were developed from specific training samples. Because these values vary with the training data and are used to calibrate the predictive models, they cannot be generalized to other samples.
