# Supplementary material for: Development, internal and external evaluation of an artificial intelligence algorithm for child growth monitoring in primary care
Source: PLOS Digit Health. 2026 Jul 15;5(7):e0001526. doi: 10.1371/journal.pdig.0001526 (PMC13372244; doi:10.1371/journal.pdig.0001526)
Supplement: S5 Table — (DOCX) [file pdig.0001526.s005.docx]

**S4 Table.** Internal evaluation: cumulative diagnostic performance of the artificial intelligence algorithm for predicting the individual risk of cases (growth hormone deficiency or Turner syndrome).

|  |  | **Pre-defined specificity** | | | | | | | | | | | | | | | | |
| --- | --- | --- | --- | --- | --- | --- | --- | --- | --- | --- | --- | --- | --- | --- | --- | --- | --- | --- |
|  |  | **>98%** | | | | |  | | | **>99%** | | | | | | | | |
| **Cumulative diagnostic performance by age category (y)** |  | **Sensitivity (N=173)** | |  | **Specificity (N=923)** | | |  | | | **Sensitivity (N=173)** | | |  | | **Specificity (N=923)** | | |
|  |  | *%  (n cases)* | *95% CI* |  | *%  (n referents)* | *95% CI* | | |  | | | *%  (n cases)* | *95% CI* | |  | | *%  (n referents)* | *95% CI* |
| 1 to <2 |  | 30.1 (52) | 23.3-37.5 |  | 97.1 (896) | 95.8-98.1 | | |  | | | 21.4 (37) | 15.5-28.3 | |  | | 98.5 (909) | 97.5-99.2 |
| 1 to <3 |  | 48.0 (83) | 40.3-55.7 |  | 96.5 (891) | 95.1-97.6 | | |  | | | 42.2 (73) | 34.7-49.9 | |  | | 98.2 (906) | 97.1-98.9 |
| 1 to <5 |  | 60.1 (104) | 52.4-67.5 |  | 95.4 (881) | 93.9-96.7 | | |  | | | 52.0 (90) | 44.3-59.7 | |  | | 97.7 (902) | 96.5-98.6 |
| 1 to <8 |  | 75.1 (130) | 68.0-81.4 |  | 94.8 (875) | 93.2-96.1 | | |  | | | 67.1 (116) | 59.5-74.0 | |  | | 97.3 (898) | 96.0-98.2 |
| 1 to <12 |  | 86.1 (149) | 80.1-90.9 |  | 94.5 (872) | 92.8-95.9 | | |  | | | 76.3 (132) | 69.3-82.4 | |  | | 97.1 (896) | 95.8-98.1 |
| **Theoretical reduction in time to diagnosis (y),** median (IQR) |  | 2.7 | 1.2-5.1 |  |  |  | | |  | | | 2.6 | 1.0-5.1 | |  | |  |  |

*CI: confidence interval, IQR: interquartile range*
