# Supplementary material for: Development, internal and external evaluation of an artificial intelligence algorithm for child growth monitoring in primary care
Source: PLOS Digit Health. 2026 Jul 15;5(7):e0001526. doi: 10.1371/journal.pdig.0001526 (PMC13372244; doi:10.1371/journal.pdig.0001526)
Supplement: S6 Table — (DOCX) [file pdig.0001526.s006.docx]

**S5 Table.** Internal evaluation: cumulative diagnostic performance of the artificial intelligence algorithm by condition.

|  |  | **Pre-defined (and calculated) specificity*** | | | | | | | | | | | | | | | | |
| --- | --- | --- | --- | --- | --- | --- | --- | --- | --- | --- | --- | --- | --- | --- | --- | --- | --- | --- |
|  |  | **>98% (94.5%)** | | | | | |  | | **>99% (97.1%)** | | | | | | | | |
|  |  | **GHD (N=86)** | |  | | **TS (N=87)** | | |  | | **GHD (N=86)** | | |  | | **TS (N=87)** | |  |
| **Sensitivities** (age 1-12 years) |  | *% (n)* | *95% CI* | |  | *% (n)* | *95% CI* | |  | | *% (n)* | *95% CI* |  | | *% (n)* | | *95% CI* |  |
|  |  | 86.0 (74) | 76.9-92.6 | |  | 86.2 (75) | 77.1-92.7 | |  | | 77.9 (67) | 67.7-86.1 |  | | 74.7 (65) | | 64.3-83.4 |  |
| **Theoretical reduction in time to diagnosis (y)**, median (IQR) |  | 1.9 | 0.7-3.5 | |  | 3.6 | 2.0-6.8 | |  | | 1.7 | 0.7-3.0 |  | | 3.9 | | 1.7-5.8 |  |

*CI: confidence interval, IQR: interquartile range, GHD: growth hormone deficiency, TS: Turner syndrome*

* The calculated specificity corresponds to the cumulative specificities calculated from referents from age 1 to 12 years during the internal evaluation (see S4 Table)
