# Supplementary material for: Development, internal and external evaluation of an artificial intelligence algorithm for child growth monitoring in primary care
Source: PLOS Digit Health. 2026 Jul 15;5(7):e0001526. doi: 10.1371/journal.pdig.0001526 (PMC13372244; doi:10.1371/journal.pdig.0001526)
Supplement: S7 Table — (DOCX) [file pdig.0001526.s007.docx]

## S6 Table. Comparison of the artificial intelligence algorithm with the multinomial logistic regression without and with Synthetic Minority Over-sampling TEchnique (SMOTE) and the extreme gradient boosting (XGBoost) regression.

|  | **Development** | | |  | **Internal evaluation** | | |  | **External evaluation** | | |
| --- | --- | --- | --- | --- | --- | --- | --- | --- | --- | --- | --- |
|  | **Multinomial** | **Multinomial with SMOTE** | **XGBoost** |  | **Multinomial** | **Multinomial with SMOTE** | **XGBoost** |  | **Multinomial** | **Multinomial with SMOTE** | **XGBoost** |
| **Pre-defined specificity > 98%** | | | | | | | | | | | |
| Sensitivity^*^, % (95% CI) | 89.6  (84.1-93.7) | 87.3  (81.4-91.9) | 95.4  (91.1-98.0) |  | 86.1  (80.1-90.9) | 86.7  (80.7-91.4) | 85.5  (79.4-90.4) |  | 84.6  (76.8-90.6) | 82.1  (73.9-88.5) | 82.9  (74.8-89.2) |
| Specificity^*^, % (95% CI) | 94.7  (93.0-96.0) | 95.0  (93.4-96.3) | 91.3  (89.3-93.1) |  | 94.5  (92.8-95.9) | 94.7  (93.0-96.0) | 93.2  (91.4-94.7) |  | 94.3  (93.6-94.9) | 95.1  (94.5-95.6) | 90.3  (89.5-91.0) |
| **Theoretical reduction in time to diagnosis (y),** median (IQR)^*^ | 2.8  (1.3-5.3) | 2.7  (1.2-5.2) | 4.3  (2.0-7.7) |  | 2.7  (1.2-5.1) | 2.6  (1.1-5.4) | 3.4  (1.5-6.7) |  | 2.0 (0.6-3.8) | 2.0  (0.6-4.1) | 2.2  (0.9-5.6) |
| **Pre-defined specificity > 99%** | | | | | | | | | | | |
| Sensitivity^*^, % (95% CI) | 82.7  (76.2-88.0) | 77.5  (70.5-83.5) | 93.1  (88.2-96.4) |  | 76.3 (69.3-82.4) | 70.5  (63.1-77.2) | 80.9  (74.3-86.5) |  | 76.9  (68.2-84.2) | 71.8  (62.7-79.7) | 78.6  (70.1-85.7) |
| Specificity^*^, % (95% CI) | 97.4  (96.2-98.3) | 97.0  (95.6-98.0) | 94.8  (93.2-96.1) |  | 97.1 (95.8-98.1) | 96.7  (95.4-97.8) | 95.4  (93.9-96.7) |  | 96.6  (96.1-97.1) | 97.2 (96.8-97.6) | 93.8  (93.1-94.4) |
| **Theoretical reduction in time to diagnosis (y),** median (IQR)^*^  **Cumulative diagnostic performance** from 1 to 12 years | 2.6  (1.1-5.0) | 2.6  (1.0-5.0) | 3.9  (1.7-7.2) |  | 2.6  (1.0-5.1) | 2.4  (0.9-4.7) | 3.1  (1.1-5.7) |  | 1.8   (0.6-3.6) | 1.6  (0.6-3.1) | 1.9  (0.8-4.8) |

*CI: confidence interval, IQR: interquartile range*

*^*^* Cumulative diagnostic performance from age 1 to 12 years
