# Supplementary material for: Development, internal and external evaluation of an artificial intelligence algorithm for child growth monitoring in primary care
Source: PLOS Digit Health. 2026 Jul 15;5(7):e0001526. doi: 10.1371/journal.pdig.0001526 (PMC13372244; doi:10.1371/journal.pdig.0001526)
Supplement: S8 Table — (DOCX) [file pdig.0001526.s008.docx]

## S7 Table. Comparison of the artificial intelligence algorithm with other existing algorithms.

|  | **Development** | | | | |  | **External evaluation** | | | | |
| --- | --- | --- | --- | --- | --- | --- | --- | --- | --- | --- | --- |
|  | Sensitivity  % (95% CI)  (N = 173) |  | Theoretical reductions in time  to diagnosis (years) median (IQR) |  | Specificity  % (95% CI)  (N=923) |  | Sensitivity  % (95% CI)  (N = 117) |  | Theoretical reductions in time  to diagnosis (years) median (IQR) |  | Specificity  % (95% CI)  (N=5,755) |
| **GHRS criteria** | 97.1 (93.4-99.1) |  | 4.3 (1.9-7.8) |  | 33.7 (30.7-36.9) |  | 96.6 (91.5-99.1) |  | 3.3 (1.2-6.8) |  | 59.8 (58.5-61.0) |
| **Grote clinical decision rule** | 81.5 (74.9-87.0) |  | 1.9 (0.5-5.4) |  | 77.0 (74.2-79.7) |  | 82.9 (74.8-89.2) |  | 1.2 (0.2-4.4) |  | 88.7 (87.9-89.6) |
| **Saari clinical decision rule**  **for Turner syndrome** | 89.6 (84.1-93.7) |  | 3.4 (1.5-6.9) |  | 25.9 (23.1-28.9) |  | 95.7 (90.3-98.6) |  | 2.9 (1.1-5.6) |  | 57.8 (56.5-59.1) |
| **Revised Coventry consensus** | 79.2 (72.4-85.0) |  | 2.1 (0.2-5.3) |  | 90.9 (88.9-92.7) |  | 81.2 (72.9-87.8) |  | 1.2 (0.2-3.2) |  | 95.7 (95.2-96.2) |
| **WHO criterion** | 88.4 (82.7-92.8) |  | 3.1 (1.1-6.3) |  | 83.1 (80.5-85.5) |  | 91.5 (84.8-95.8) |  | 2.1 (0.8-5.1) |  | 90.2 (89.4-91.0) |
|  |  |  |  |  |  |  |  |  |  |  |  |
| **AI algorithm**  Pre-defined (and calculated) specificity 98% | 89.6 (84.1-93.7)^**^ |  | 2.8 (1.3-5.3) |  | 94.7 (93.0-96.0)^**^ |  | 84.6 (76.8-90.6) |  | 2.0 (0.6-3.8) |  | 94.3 (93.6-94.9)^***^ |
| **AI algorithm**  Pre-defined (and calculated) specificity 99% | 82.7 (76.2-88.0) |  | 2.6 (1.1-5.0) |  | 97.4 (96.2-98.3)^***^ |  | 76.9 (68.2-84.2) |  | 1.8 (0.6-3.6) |  | 96.6 (96.1-97.1)^**^ |

*CI: confidence interval, IQR: interquartile range; GHRS: Growth Hormone Research Society.*

Statistically significant difference in sensitivity or specificity between the revised Coventry consensus and AI algorithm according to the results of the McNemar test for pairs (^*^p < 0.05, ^**^p < 0.01, ^***^p < 0.001).
