# Supplementary material for: Development, internal and external evaluation of an artificial intelligence algorithm for child growth monitoring in primary care
Source: PLOS Digit Health. 2026 Jul 15;5(7):e0001526. doi: 10.1371/journal.pdig.0001526 (PMC13372244; doi:10.1371/journal.pdig.0001526)
Supplement: S1 Fig — (DOCX) [file pdig.0001526.s009.docx]

**S1 Fig.** Running the Jenss-Bayley model for sequentially modeling the height growth curve of a child and application of an artificial intelligence algorithm.

An example of an observed height growth curve (black solid line) for a child with Turner syndrome diagnosed at age 116.5 months. The growth curves were sequentially modeled at each visit by using the Jenss-Bayley model (illustrated in blue solid line). The curves are superimposed on the mean height growth curves for referents (illustrated in green solid line) and cases (illustrated in red solid line), and the French height growth references (illustrated in blue dashed line). From age 1 year, the algorithm was applied at each visit of this child, and the algorithm became positive first at age 24 months, for a theoretical reduction in time to diagnosis of 7.7 years. TS, Turner syndrome

*
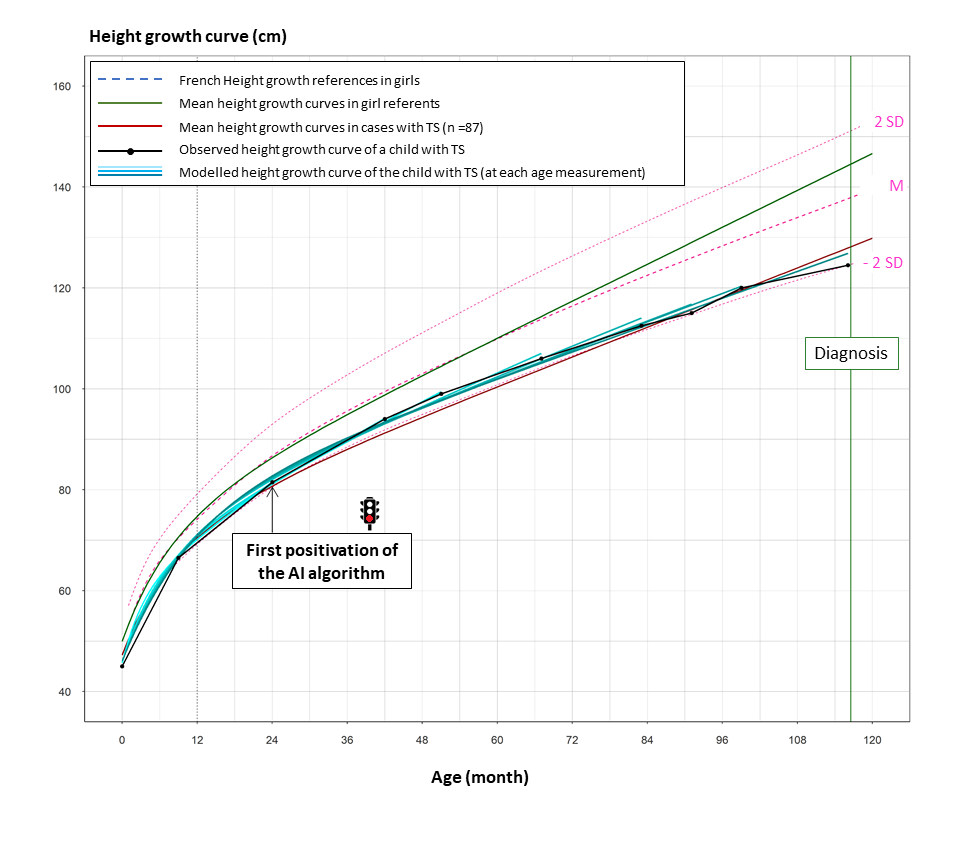
*
