# Supplementary material for: Development, internal and external evaluation of an artificial intelligence algorithm for child growth monitoring in primary care
Source: PLOS Digit Health. 2026 Jul 15;5(7):e0001526. doi: 10.1371/journal.pdig.0001526 (PMC13372244; doi:10.1371/journal.pdig.0001526)
Supplement: S2 Fig — (DOCX) [file pdig.0001526.s010.docx]

**S2 Fig.** Development: multicollinearity maps.

A Spearman correlation coefficient > 0.80 and a variance inflation factor (VIF) > 5 (equivalent to tolerance < 0.2) indicated strong multicollinearity.

| **1 to <2 years** | **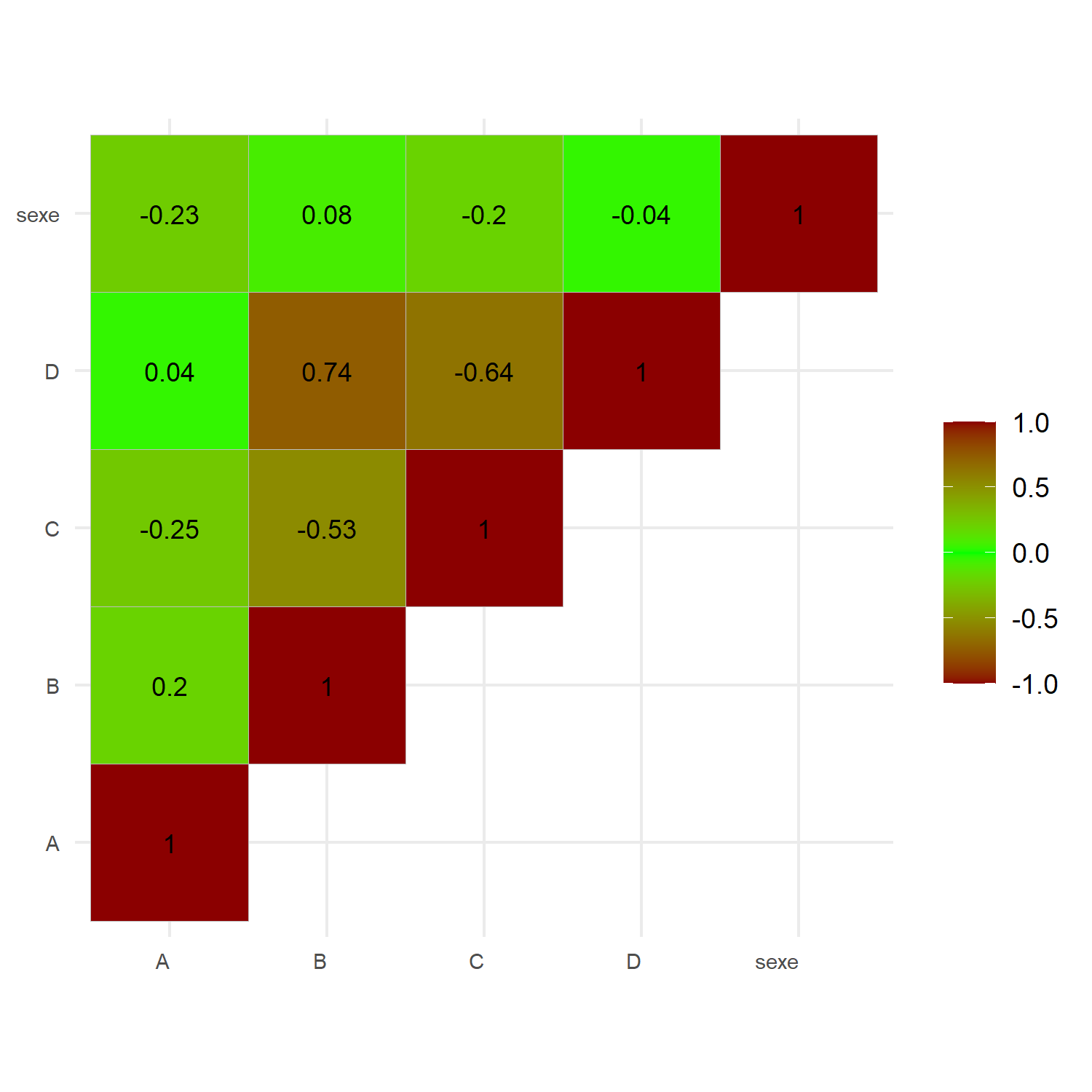** | **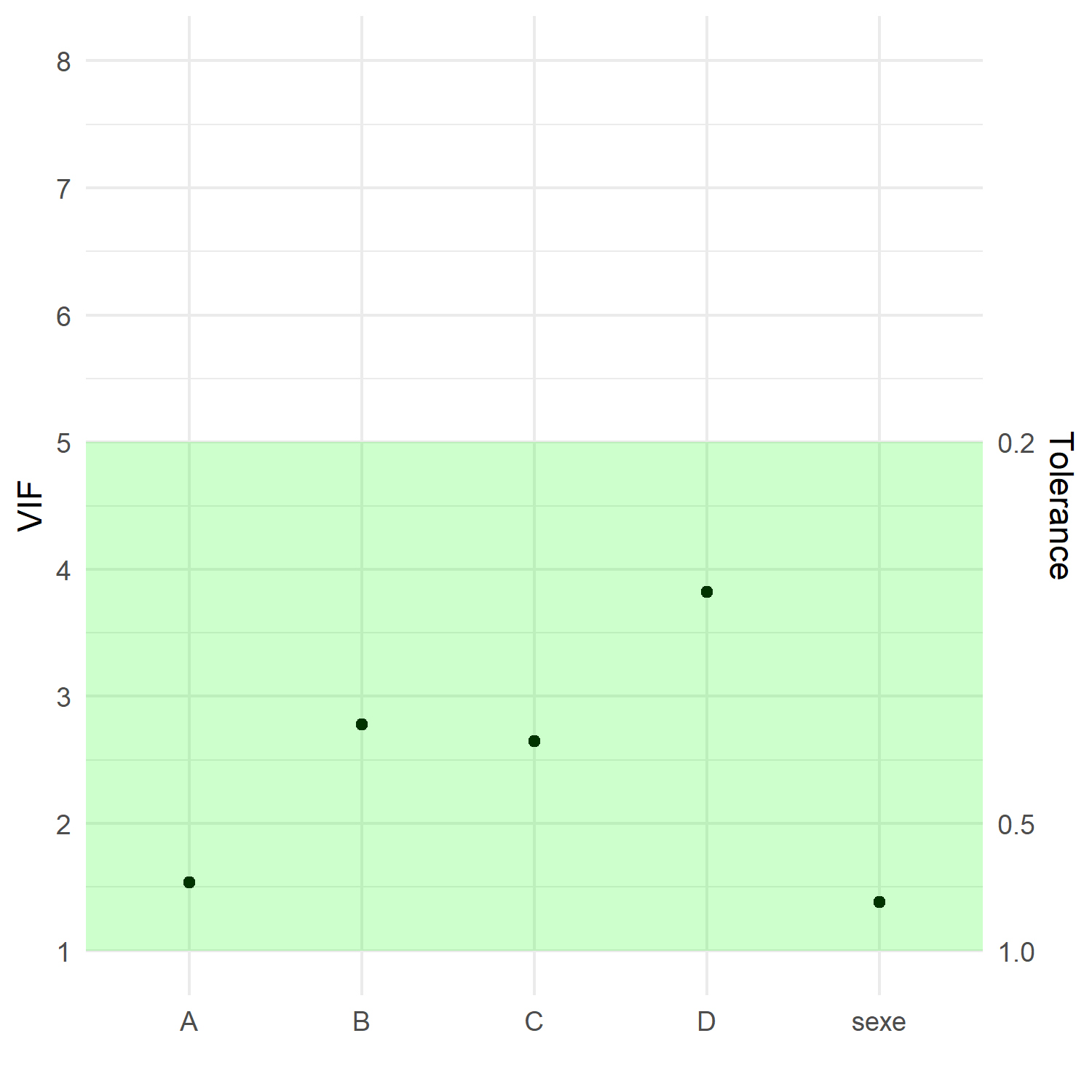** |
| --- | --- | --- |
| **2 to <3 years** | **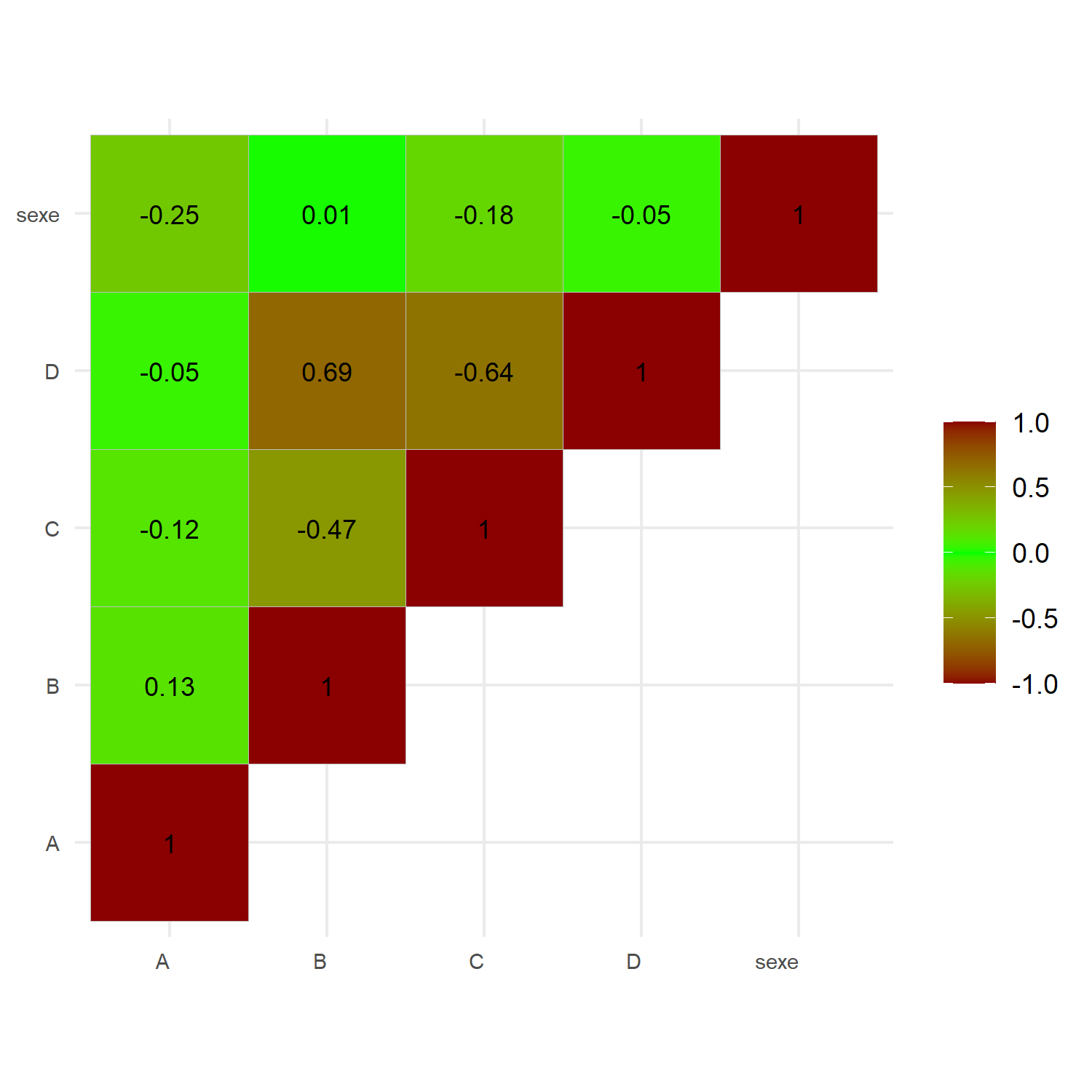** | **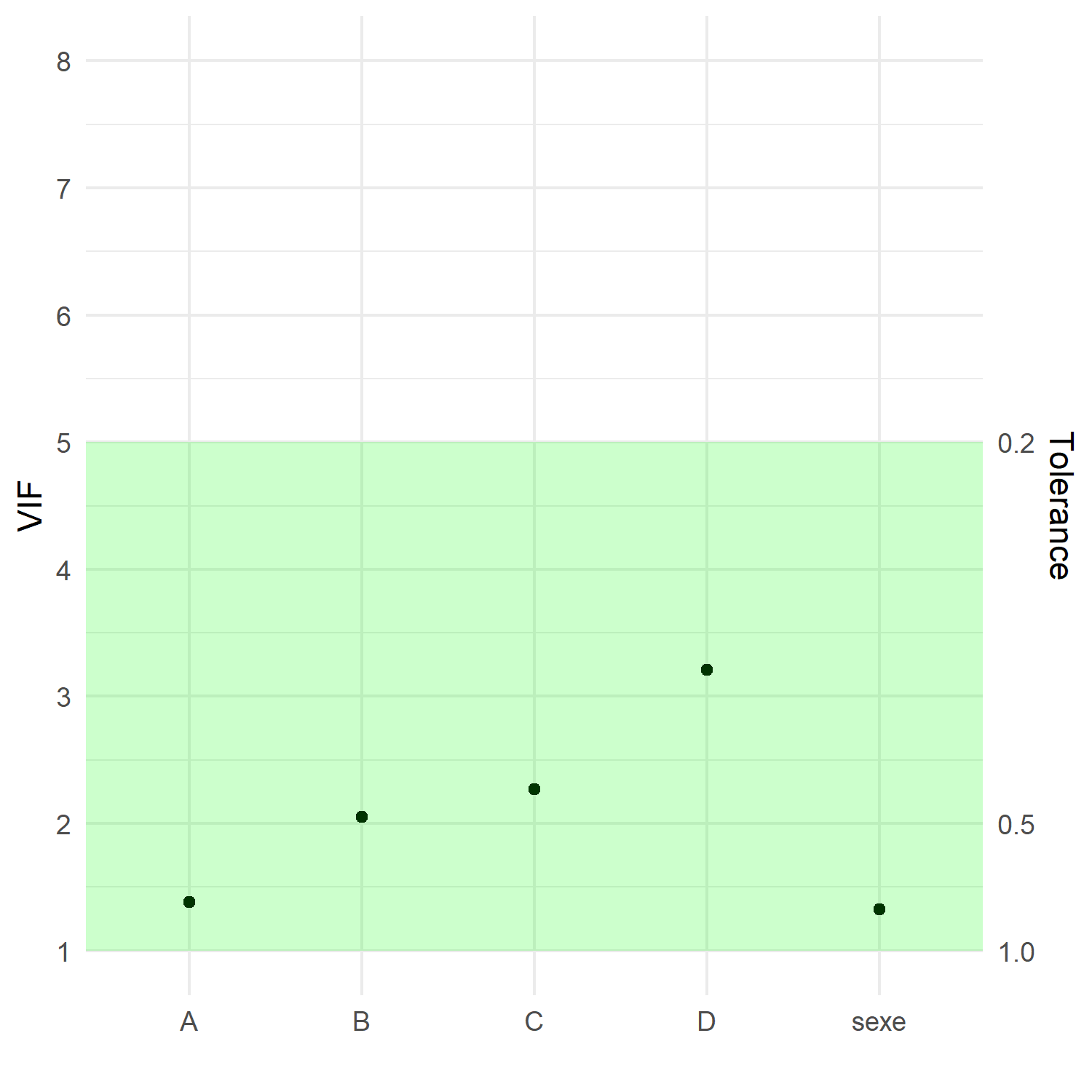** |
| **3 to <5 years** | **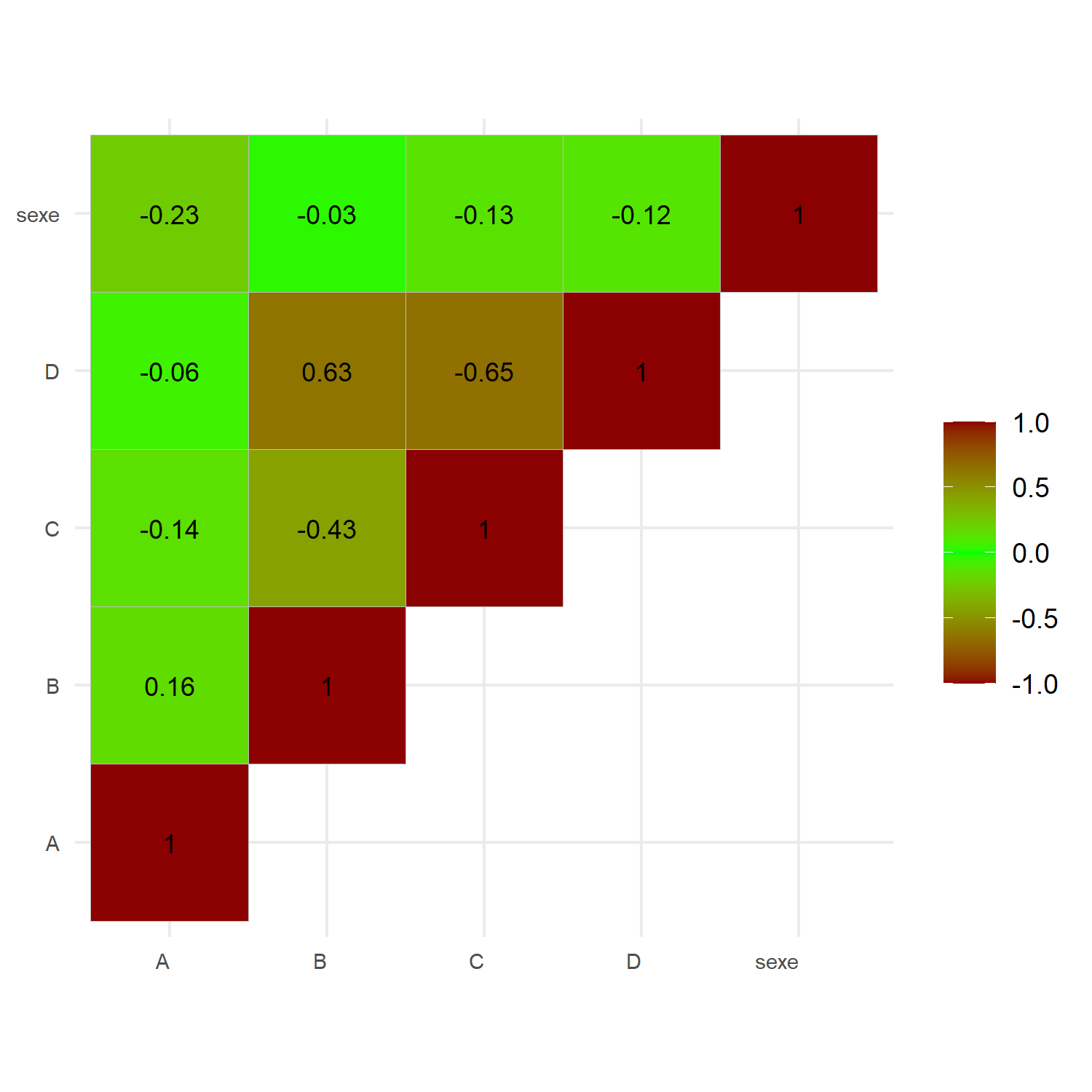** | **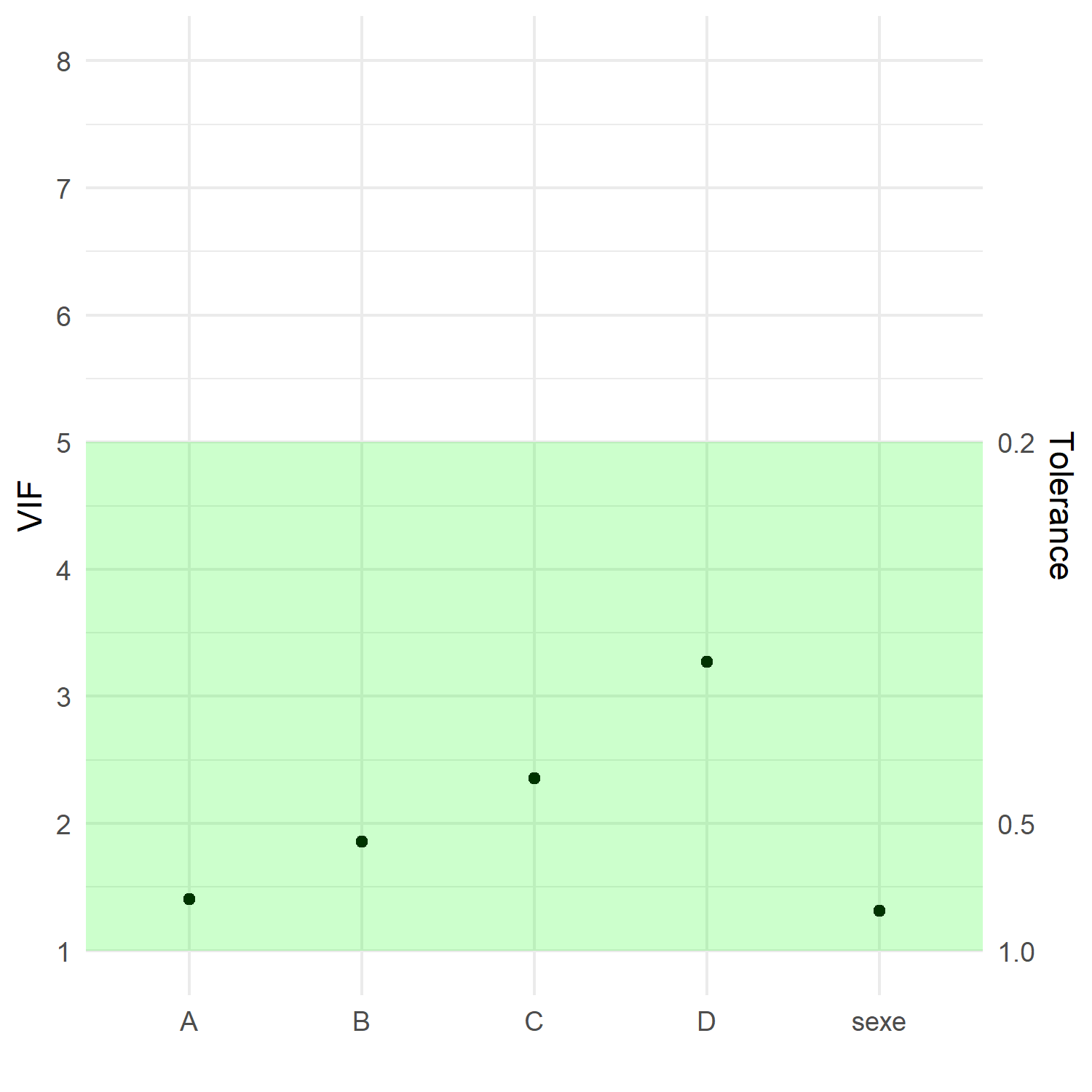** |
| **5 to <8 years** | **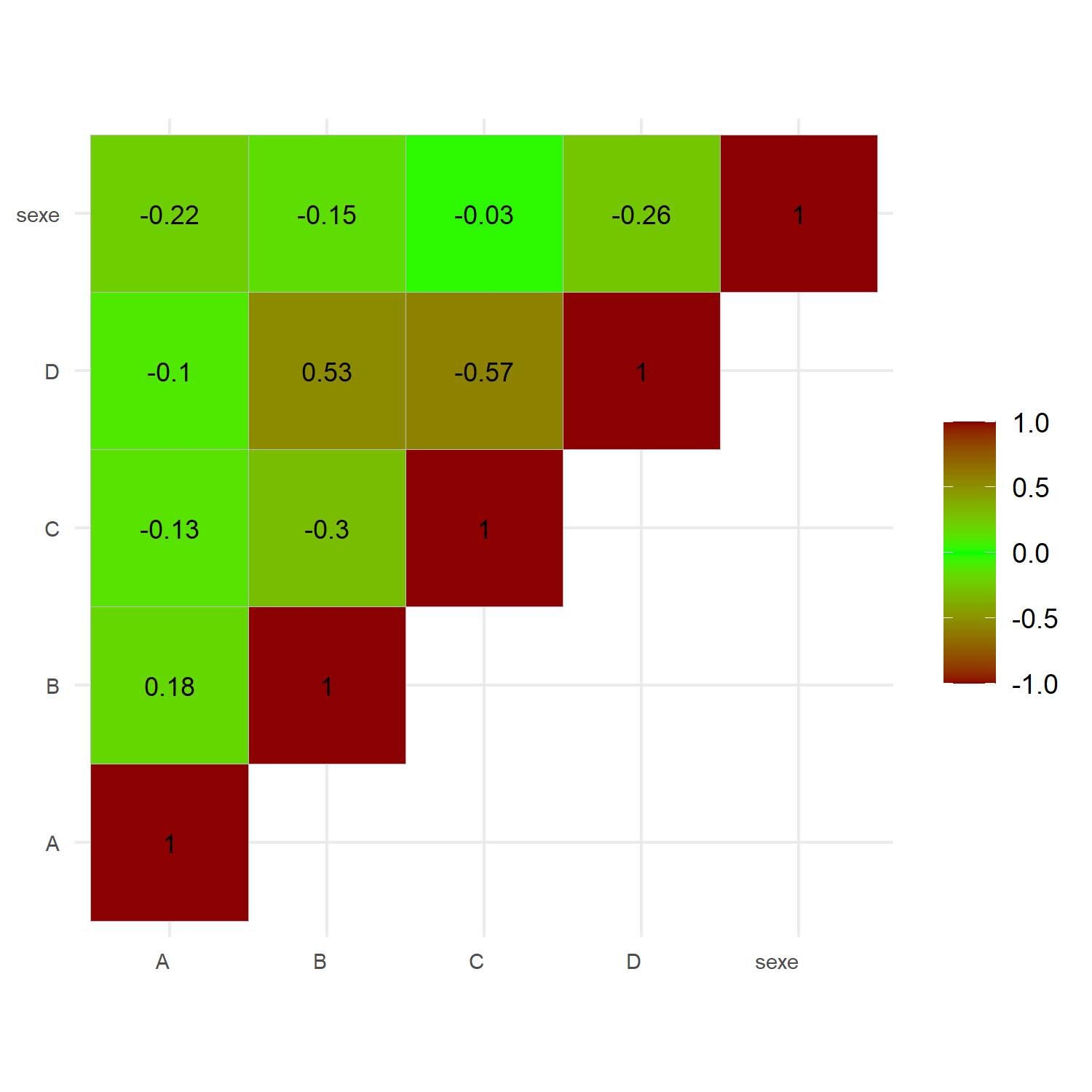** | **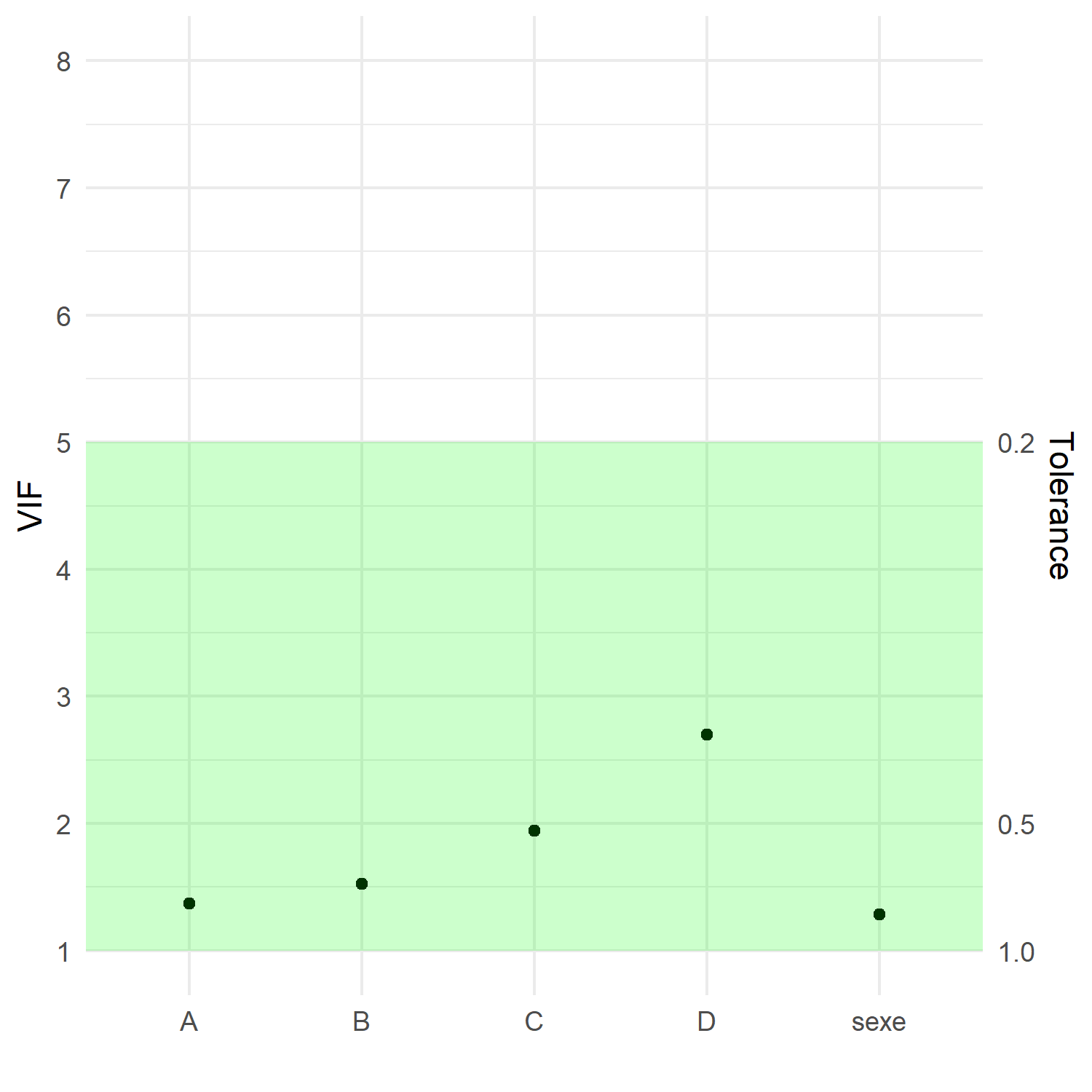** |
| **8 to <12 years** | **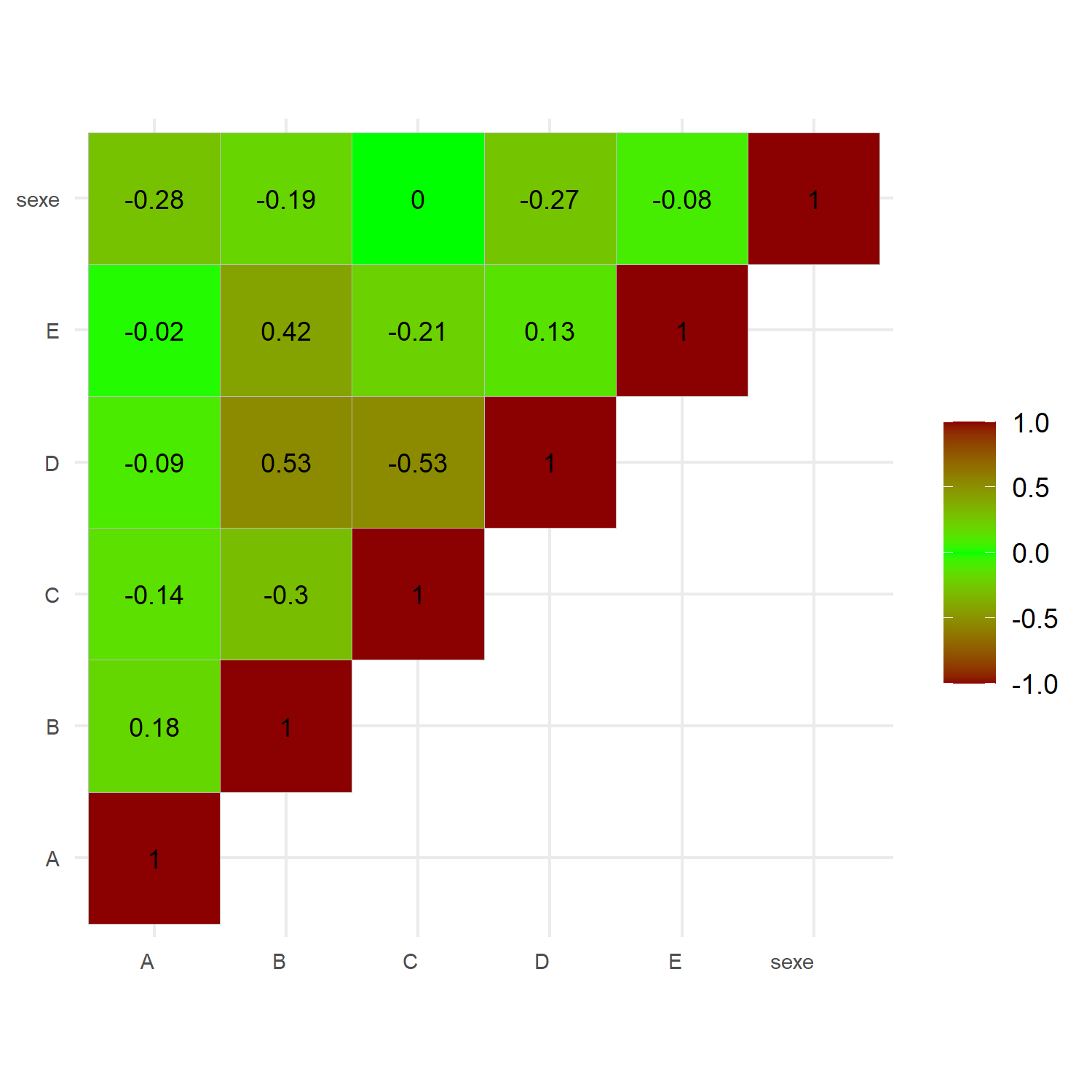** | **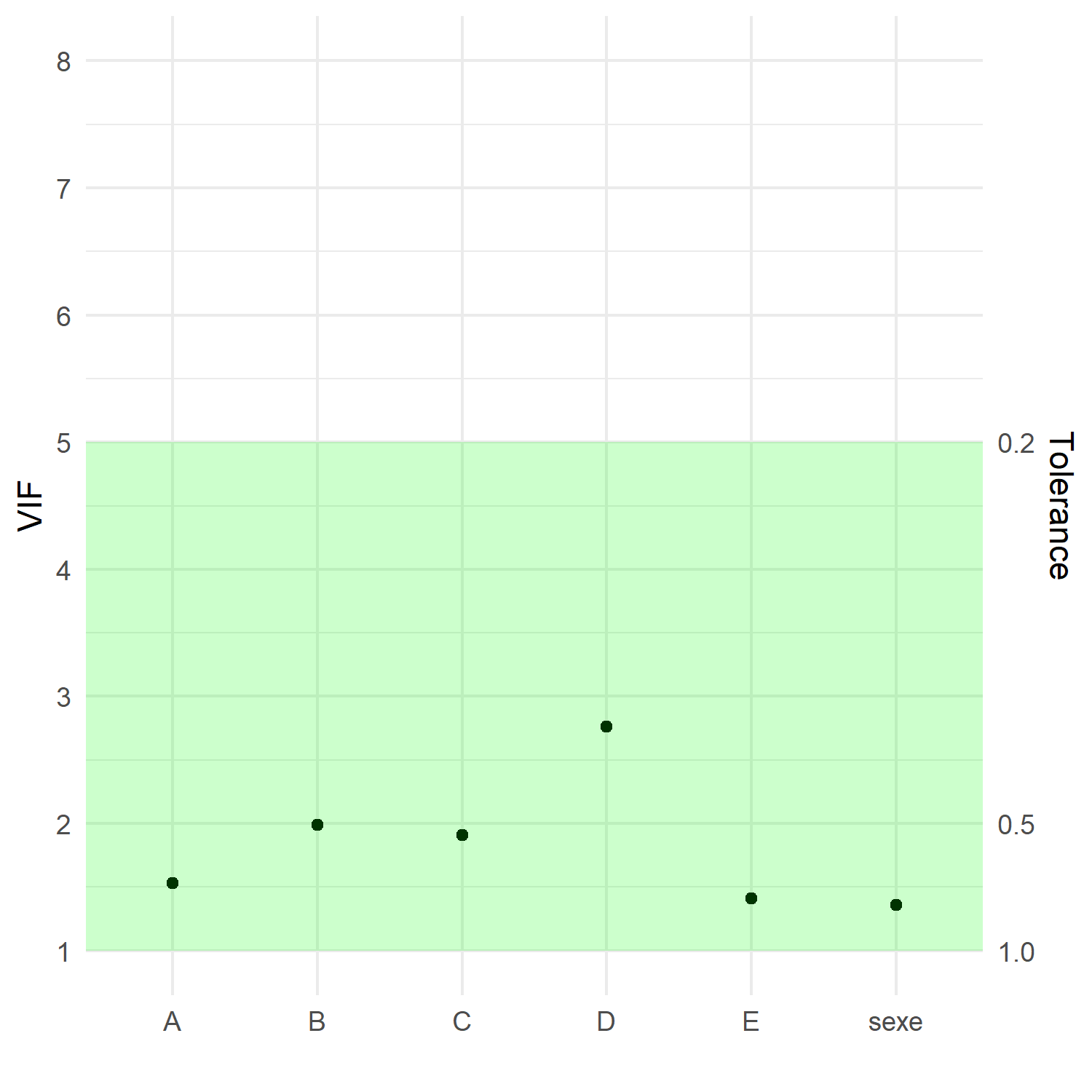** |
