# Supplementary material for: Development, internal and external evaluation of an artificial intelligence algorithm for child growth monitoring in primary care
Source: PLOS Digit Health. 2026 Jul 15;5(7):e0001526. doi: 10.1371/journal.pdig.0001526 (PMC13372244; doi:10.1371/journal.pdig.0001526)
Supplement: S3 Fig — (DOCX) [file pdig.0001526.s011.docx]

**S3 Fig.** Development: distribution of the five growth parameters, from the Jenss-Bayley model, for growth hormone deficiency (GHD) cases, Turner syndrome cases, and referents from birth to age 12 years, for girls and boys.

| **Girls**  **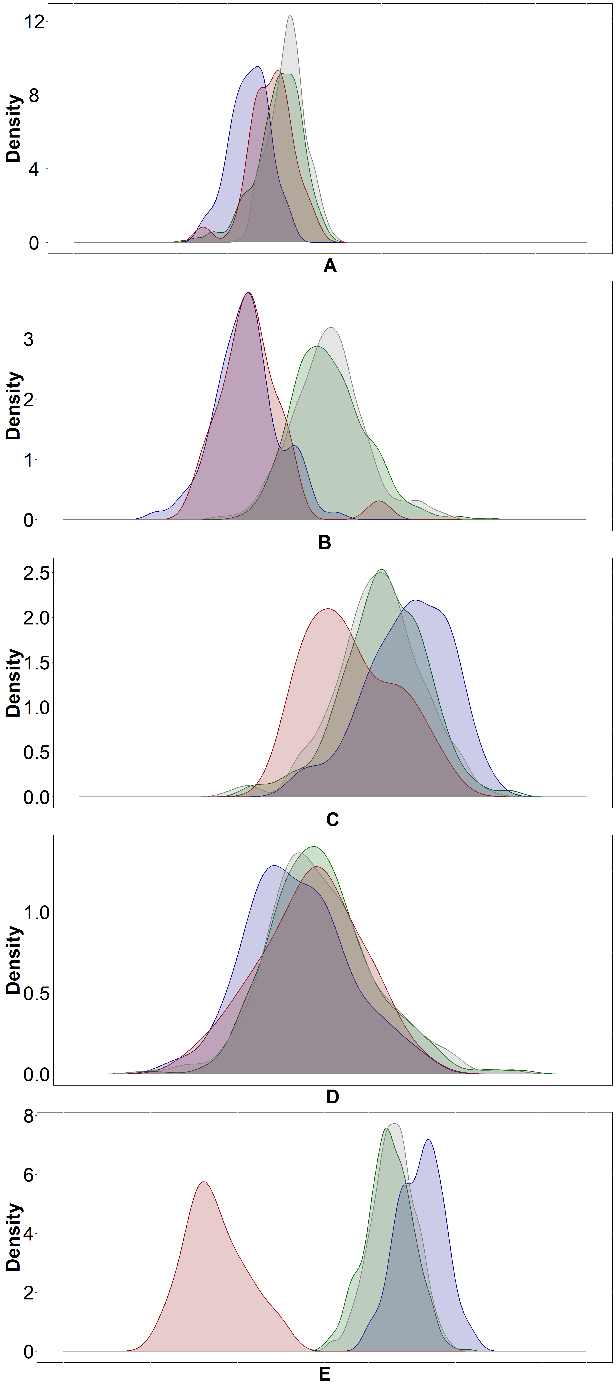** | **Boys**  **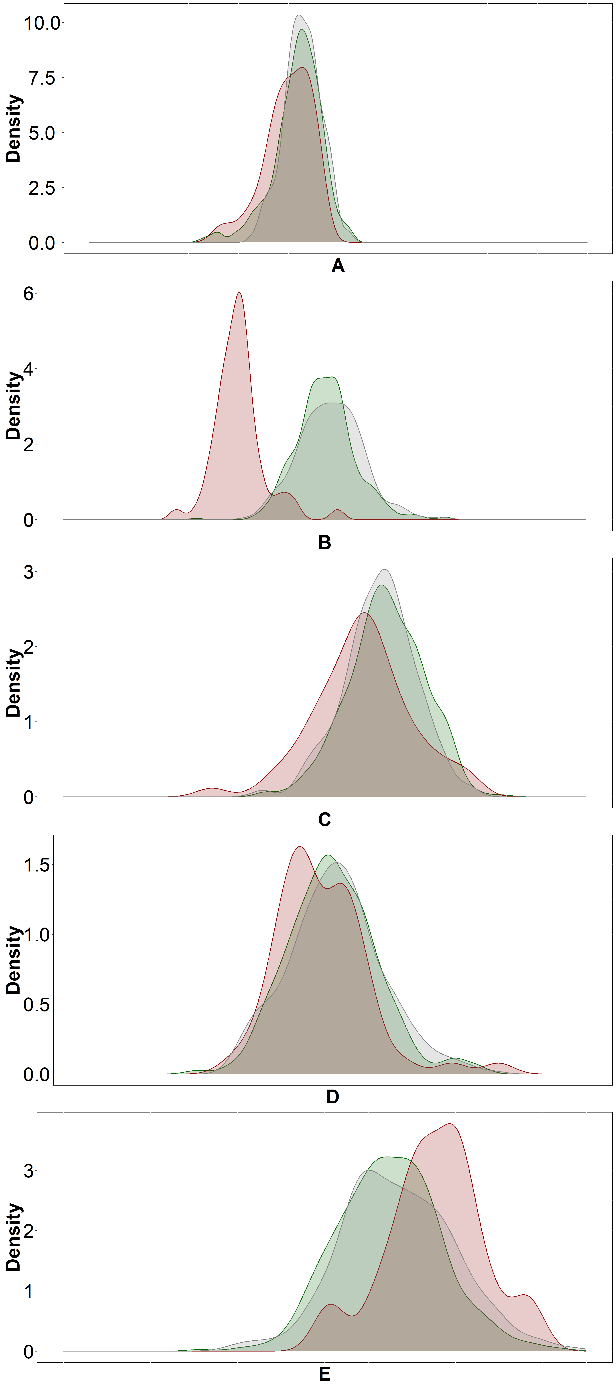** |
| --- | --- |
| 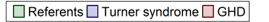 | |
