# Supplementary material for: Development, internal and external evaluation of an artificial intelligence algorithm for child growth monitoring in primary care
Source: PLOS Digit Health. 2026 Jul 15;5(7):e0001526. doi: 10.1371/journal.pdig.0001526 (PMC13372244; doi:10.1371/journal.pdig.0001526)
Supplement: S4 Fig — (DOCX) [file pdig.0001526.s012.docx]

**S1 Fig.** Internal evaluation: area under the receiver operating characteristic curves (AUROCs) for age-specific predictive models.

| 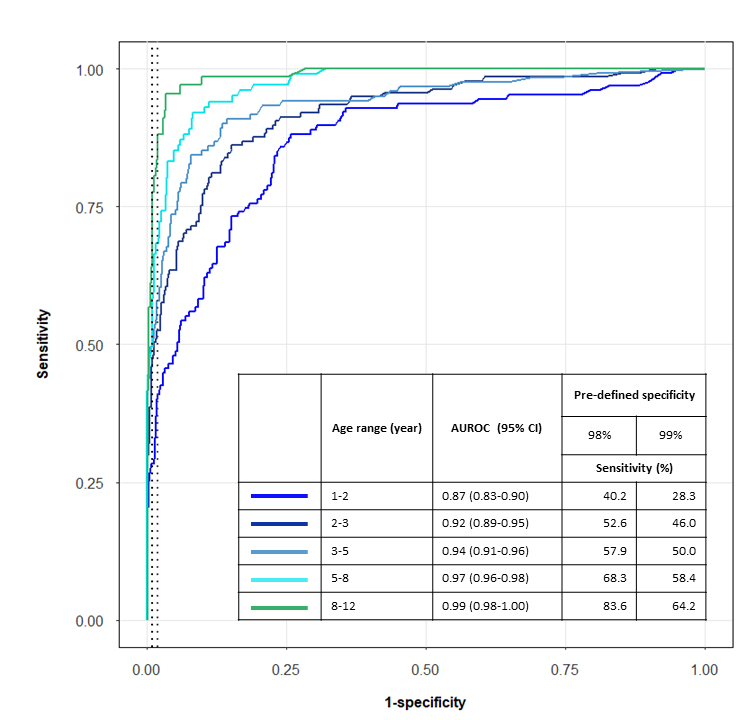 |
| --- |

*CI: confidence interval.*
