# Supplementary figures and images for: Development, internal and external evaluation of an artificial intelligence algorithm for child growth monitoring in primary care
Source: PLOS Digit Health. 2026 Jul 15;5(7):e0001526. doi: 10.1371/journal.pdig.0001526 (PMC13372244; doi:10.1371/journal.pdig.0001526)

**S5 Fig.** Internal evaluation: calibration curves for age-specific predictive models.


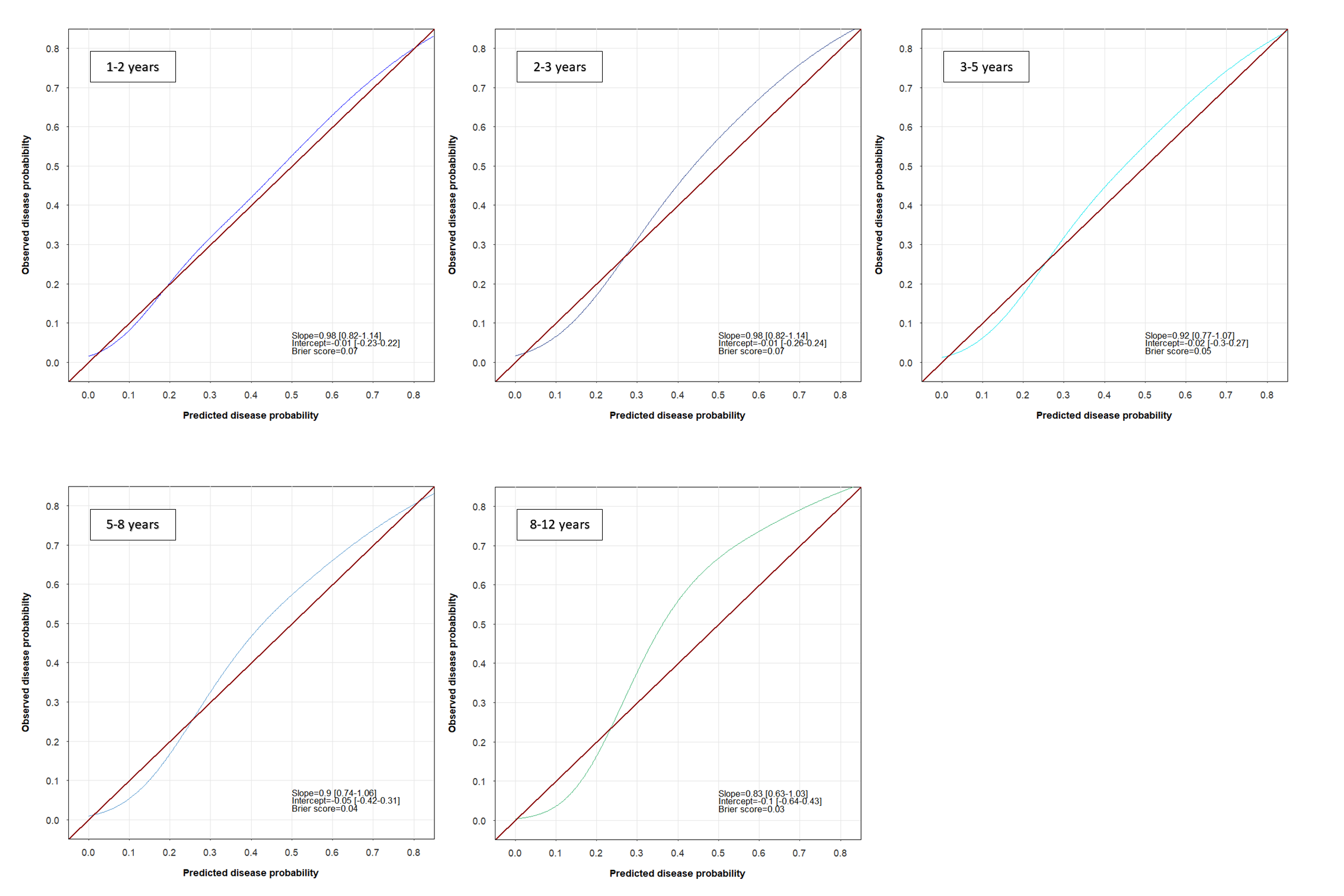

Supplement: S5 Fig — (DOCX) [file pdig.0001526.s013.docx]

**S6 Fig.** Internal evaluation: decision curve analysis for age-specific predictive models.


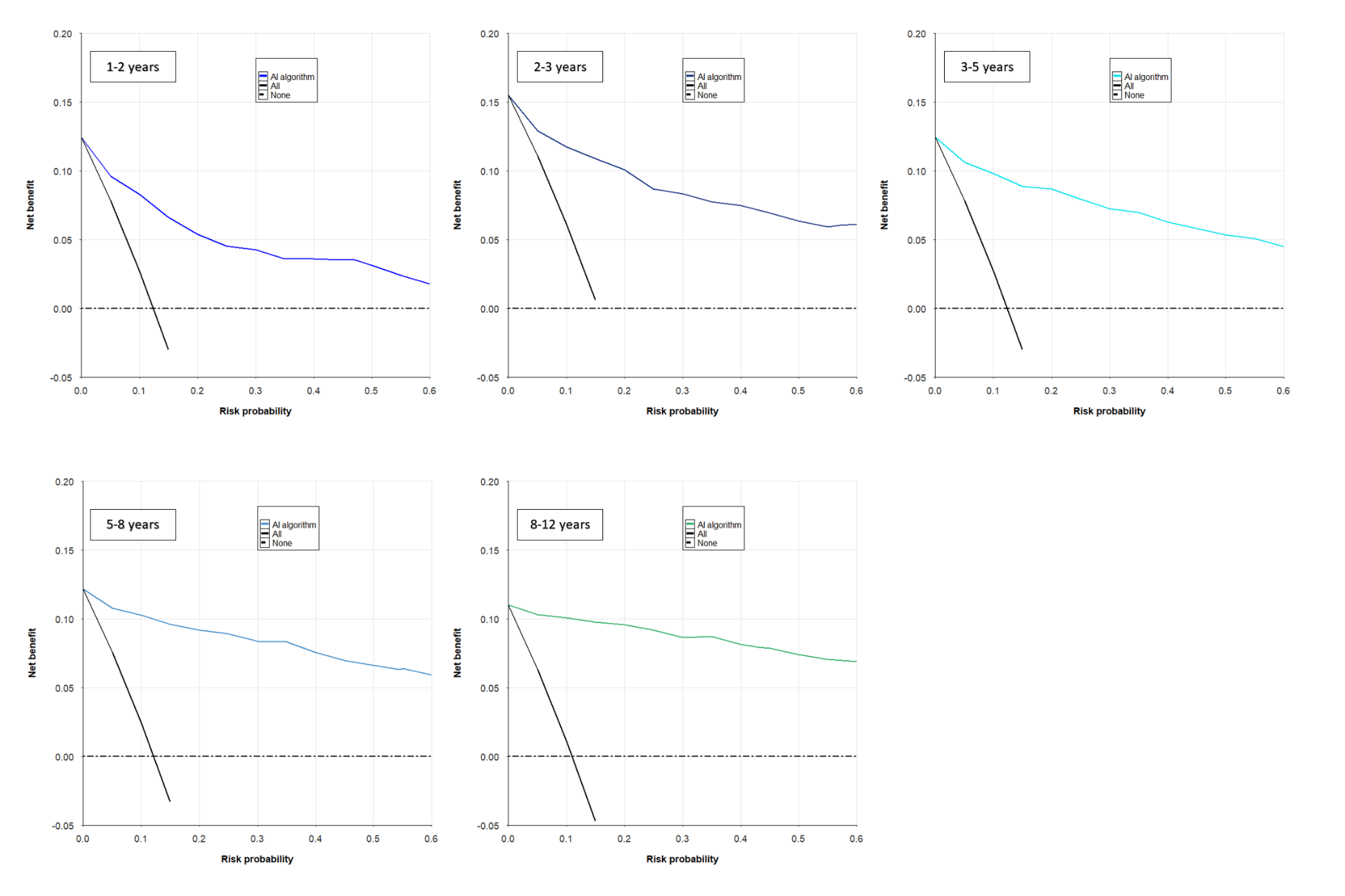

Supplement: S6 Fig — (DOCX) [file pdig.0001526.s014.docx]
