# Supplementary material for: Development, internal and external evaluation of an artificial intelligence algorithm for child growth monitoring in primary care
Source: PLOS Digit Health. 2026 Jul 15;5(7):e0001526. doi: 10.1371/journal.pdig.0001526 (PMC13372244; doi:10.1371/journal.pdig.0001526)
Supplement: S1 Box — (DOCX) [file pdig.0001526.s015.docx]

**S1 Box.** Identification of cases and data extraction from the clinical data warehouse.

To identify diseased children with a diagnosis of growth hormone deficiency (GHD) or Turner syndrome (TS) in the Assistance Publique-Hôpitaux de Paris (AP-HP) clinical data warehouse, we used specific discharge codes for GHD (E230, E236) or TS (Q96), according to the International Classification of Diseases, 10^th^ Revision. To confirm the definitive diagnosis of children, we conducted a systematic and automatic review of their electronic health records within this clinical data warehouse using a natural language processing algorithm based on relevant terms related to GHD or TS, followed by a manual confirmation by one author. We then selected the children with a karyotype for TS and an MRI scan showing a pituitary stalk interruption syndrome for GHD. We extracted baseline data (sex, gestational age, birth weight and length, parental height, date of diagnosis) and height measurements from electronic health records of children using the REDCap software (<https://www.project-redcap.org/>).
