# Supplementary material for: Development, internal and external evaluation of an artificial intelligence algorithm for child growth monitoring in primary care
Source: PLOS Digit Health. 2026 Jul 15;5(7):e0001526. doi: 10.1371/journal.pdig.0001526 (PMC13372244; doi:10.1371/journal.pdig.0001526)
Supplement: S2 Box — (DOCX) [file pdig.0001526.s016.docx]

**S2 Box.** Mixed-effect Jenss-Bayley model.^1-3^

The equation to model height Y_ij_ (in cm) of child i at age t_ij_ (in days) can be expressed as:

| $Y_{\mathrm{ij}}= Ai+Bi\times t_{\mathrm{ij}}+e^{(\mathrm{Ci})}\times\left( 1 -e^{Di \times t_{\mathrm{ij}}} \right)+({Ei \times t_{\mathrm{ij}}}^{2}){+ \varepsilon}_{\mathrm{ij}}$ |
| --- |

Height growth curves of children were determined according to five growth parameters: $A_{i}$, $B_{i}$, $C_{i}$ , $D_{i}$, and $E_{i}$. To improve model convergence, we applied constraints by applying the exponential function to the growth parameters. Birth height ($A_{i}$) and curve slope after age 2 years ($B_{i}$) are constrained to be positive; the spurt of growth between 0 and 2 years ($C_{i}$) must also be positive because the growth velocity from 1 year onward is lower than the growth velocity in the first month of life; the pre-pubertal height acceleration after age 8 years (Ei) is constrained to be positive; and the curvature of the trajectory between 0 and 2 years ($D_{i}$) must be negative because it reflects decrease of growth velocity during the first months of life*.^4^* Random effects terms were added to the model for each growth parameter to account for between-individual variability. We included interaction terms with a covariable (Z), which corresponds to the clinical status of the child (i.e., GHD case, TS case, or referent).

| $Y_{\mathrm{ij}}= e^{(A_{i})}+e^{(B_{i})}\times t_{\mathrm{ij}}+e^{\left( C_{i} \right)}\times\left( 1 -e^{{-e}^{\left( D_{i} \right)} \times t_{\mathrm{ij}}} \right)+{{(e}^{(E_{i})} \times t_{\mathrm{ij}}}^{2}){+ \varepsilon}_{\mathrm{ij}}$ |
| --- |
| $A_{i}=\alpha_{A}+ \gamma_{A.TS}*Z_{TS,i}+ \gamma_{A.GHD}*Z_{GHD,i}+u_{A_{i}}$  $B_{i}=\alpha_{B}+\gamma_{B.TS}*Z_{TS,i}+ \gamma_{B.GHD}*Z_{GHD,i}+u_{B_{i}}$  $C_{i}=\alpha_{C}+ \gamma_{C.TS}*Z_{TS,i}+ \gamma_{C.GHD}*Z_{GHD,i}+u_{C_{i}}$  $D_{i}=\alpha_{D}+\gamma_{D.TS}*Z_{TS,i}+ \gamma_{D.GHD}*Z_{GHD,i}+u_{D_{i}}$  $E_{i}=\alpha_{E}+ \gamma_{E.TS}*Z_{TS,i}+ \gamma_{E.GHD}*Z_{GHD,i}+u_{E_{i}}$  $\alpha_{A},\alpha_{B},\alpha_{C},\alpha_{D},\alpha_{E} represent the fixed effect ofA, B, C, D, E (when Z='referents')$  $\gamma_{A}, \gamma_{B}, \gamma_{C}, \gamma_{D}, \gamma_{E} represent the interaction with clinical status \left( Z \right) (ref='referents')$  $u_{A_{i}}, u_{B_{i}}, u_{C_{i}}, u_{D_{i}}, u_{E_{i}}are the individual random effect of A, B, C, D, E$  $\varepsilon_{ij} is the individual residual error$ |
| $u_{i}= \left( \begin{matrix} u_{A_{i}} \\ u_{B_{i}} \\ u_{C_{i}} \\ u_{D_{i}} \\ u_{E_{i}} \end{matrix} \right)\sim N\left( \left[ \begin{matrix} 0 \\ 0 \\ 0 \\ 0 \\ 0 \end{matrix} \right], \left[ \begin{matrix} \sigma_{u_{A_{i}}}^{^{2}} & \mathrm{cov}_{{(u}_{A_{i}},u_{B_{i)}}} & \ldots& \ldots& \ldots\\ \ldots& \sigma_{u_{B_{i}}}^{^{2}} & \ldots& \ldots& \ldots\\ \ldots& \ldots& \sigma_{u_{C_{i}}}^{^{2}} & \ldots& \ldots\\ \ldots& \ldots& \ldots& \sigma_{u_{D_{i}}}^{^{2}} & \ldots\\ \ldots& \ldots& \ldots& \ldots& \sigma_{u_{E_{i}}}^{^{2}} \end{matrix} \right] \right)$ $\varepsilon_{\mathrm{ij}} \sim N(0, \sigma_{e}^{2})$ |
| 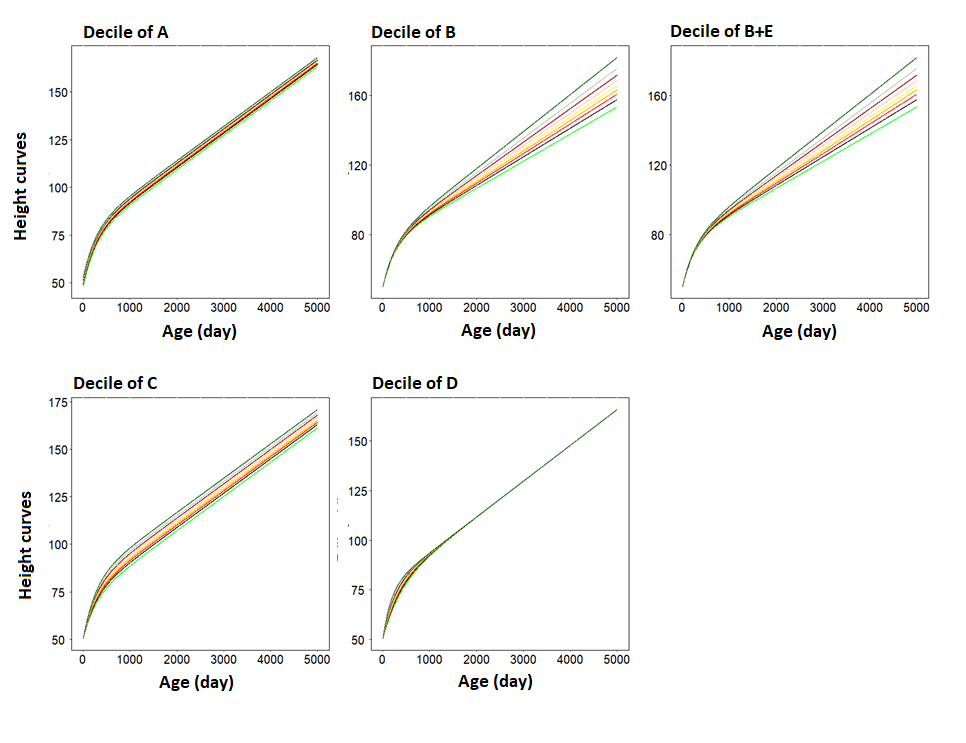 |

The Stochastic Approximation Expectation Maximization (SAEM) algorithm combined with Markov chain Monte Carlo (MCMC) methods was used for estimating model coefficients for maximizing the log-likelihood. A non-structured variance-covariance matrix was applied to account for the correlation between growth parameters. The residual variability was modeled by using a proportional heteroscedastic error model structure. Model adequacy was assessed by analyzing residual plots (heteroscedastic and normality).

**References**

1. Botton J, Heude B, Maccario J, Borys JM, Lommez A, Ducimetiere P, et al. Parental body size and early weight and height growth velocities in their offspring. Early Hum Dev. 2010;**86**:445-50.

2. Botton J, Heude B, Maccario J, Ducimetière P, Charles MA. Postnatal weight and height growth velocities at different ages between birth and 5 y and body composition in adolescent boys and girls. Am J Clin Nutr. 2008;**87**:1760-8.

3. Botton J, Scherdel P, Regnault N, Heude B, Charles MA, Eden Mother-Child Cohort Study Group. Postnatal weight and height growth modeling and prediction of body mass index as a function of time for the study of growth determinants. Ann Nutr Metab. 2014;**65**:156-66.

4. Carles S, Charles MA, Forhan A, Slama R, Heude B, Botton J, et al. A novel method to describe early offspring Body Mass Index (BMI) trajectories and to study its determinants. PLoS One. 2016;**11**:e0157766.
