# Supplementary material for: Development, internal and external evaluation of an artificial intelligence algorithm for child growth monitoring in primary care
Source: PLOS Digit Health. 2026 Jul 15;5(7):e0001526. doi: 10.1371/journal.pdig.0001526 (PMC13372244; doi:10.1371/journal.pdig.0001526)

**S3 Box.** Age-range specific predictive models: development and evaluation.

Development

We developed the artificial intelligence (AI)-based algorithm using multinomial logistic regression, which allows for handling multiple Y classes and offers the possibility of calculating predictions for each targeted condition (with class probabilities summing to 1) and a unique threshold, resulting in a specificity of > 98% or 99%. We built five age-specific predictive models according to the equation below from the entire dataset.

| $logit(P\left( Y_{i}=k_{i} \vert X_{i} \right))={\beta_{0}+\beta_{1}A}_{i}+{\beta_{2}B}_{i}+{\beta_{3}C}_{i}+{\beta_{4}D}_{i}+\beta_{5}\mathrm{sex}_{i} \left( if age<8 years \right)$  $logit(P\left( Y_{i}=k_{i} \vert X_{i} \right))={\beta_{0}+\beta_{1}A}_{i}+{\beta_{2}B}_{i}+{\beta_{3}C}_{i}+{\beta_{4}D}_{i}+{\beta_{5}E}_{i}+\beta_{6}\mathrm{sex}_{i} \left( if age \geq8 years \right)$  Where $k_{i}$:$\left\{ \begin{aligned} 0:referents \\ 1:TS \\ 2:GHD \end{aligned} \right.$ for a child i |
| --- |

The hyperparameter tuned was the decay parameter (L2 regulation), testing different values (from 0 to 1). The optimal decay was identified by log-loss metric performance and validated by using resampling techniques (five-fold cross-validation, repeated 1 time). The Broyden–Fletcher–Goldfarb–Shanno solver (by default) was used for estimating model coefficients for maximizing the log-likelihood with a maximum of 1000 iterations to ensure convergence. Model adequacy was assessed by analysing residual plots (heteroscedastic and normality).

Internal evaluation using K-fold cross-validation

We evaluated predictive models by dividing the entire dataset into randomly selected training (9/10) and validation (1/10) sets and repeated that process 10 times, rotating the validation set. By fold, the model was trained from each training set and applied to each validation set for which risk prediction of having growth hormone deficiency (GHD) or Turner syndrome (TS) had been obtained. We combined all individual prediction risks obtained from each validation set. We dichotomized disease status (diseased vs non-diseased) and risk predictions from a system scoring based on the prediction risks for classifying the children as diseased (positive) or non-diseased (negative) by summing the risk prediction of having GHD or TS and obtaining an overall risk prediction of disease:

| If a child had $\sum(logit(P\left( \mathrm{yi}=1 \vert\mathrm{Xi} \right)), logit(P \left( \mathrm{yi}=2 \vert\mathrm{Xi} \right)))>r\mathrm{isk} \mathrm{threshold}$ then classified as “positive” else ‘negative” |
| --- |


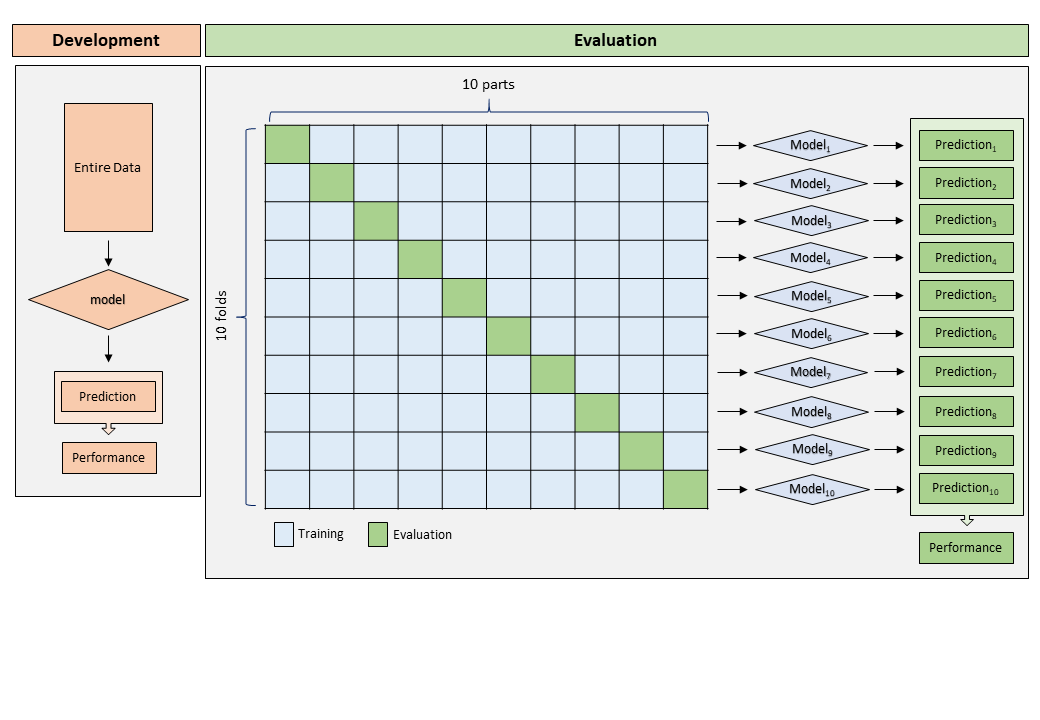

Supplement: S3 Box — (DOCX) [file pdig.0001526.s017.docx]
